# A nanoreactor boosts chemodynamic therapy and ferroptosis for synergistic cancer therapy using molecular amplifier dihydroartemisinin

**DOI:** 10.1186/s12951-022-01455-0

**Published:** 2022-05-14

**Authors:** Xiao-Xin Yang, Xiang Xu, Mei-Fang Wang, Hua-Zhen Xu, Xing-Chun Peng, Ning Han, Ting-Ting Yu, Liu-Gen Li, Qi-Rui Li, Xiao Chen, Yu Wen, Tong-Fei Li

**Affiliations:** 1 School of Basic Medical Sciences, Hubei University of Medicine, Renmin Road No. 30, Shiyan, 442000 Hubei China; 2 Hubei Key Laboratory of Embryonic Stem Cell Research, Taihe Hospital of Shiyan, Hubei University of Medicine, Renmin Road No. 30, Shiyan, 442000 Hubei China; 3 School Institute of Chemical Biology and Nanomedicine, State Key Laboratory of Chemo/Biosensing and Chemometrics, College of Chemistry and Chemical Engineering, Hunan University, Changsha, 410082 Hunan China; 4 School of Materials Science and Engineering, Central South University, Changsha, 410083 Hunan China; 5 Department of Pharmacology, School of Basic Medical Sciences, Wuhan University, Donghu Avenue No.185, Wuhan, 430072 China; 6 Hubei Provincial Key Laboratory of Developmentally Originated Disease, Wuhan, 430071 China

**Keywords:** Chemodynamic therapy (CDT), Nanoreactor, Dihydroartemisinin (DHA), Nanoscale metal–organic framework (nMOF), Ferroptosis

## Abstract

**Background:**

Chemodynamic therapy (CDT) relying on intracellular iron ions and H_2_O_2_ is a promising therapeutic strategy due to its tumor selectivity, which is limited by the not enough metal ions or H_2_O_2_ supply of tumor microenvironment. Herein, we presented an efficient CDT strategy based on Chinese herbal monomer-dihydroartemisinin (DHA) as a substitute for the H_2_O_2_ and recruiter of iron ions to amplify greatly the reactive oxygen species (ROS) generation for synergetic CDT-ferroptosis therapy.

**Results:**

The DHA@MIL-101 nanoreactor was prepared and characterized firstly. This nanoreactor degraded under the acid tumor microenvironment, thereby releasing DHA and iron ions. Subsequent experiments demonstrated DHA@MIL-101 significantly increased intracellular iron ions through collapsed nanoreactor and recruitment effect of DHA, further generating ROS thereupon. Meanwhile, ROS production introduced ferroptosis by depleting glutathione (GSH), inactivating glutathione peroxidase 4 (GPX4), leading to lipid peroxide (LPO) accumulation. Furthermore, DHA also acted as an efficient ferroptosis molecular amplifier by direct inhibiting GPX4. The resulting ROS and LPO caused DNA and mitochondria damage to induce apoptosis of malignant cells. Finally, in vivo outcomes evidenced that DHA@MIL-101 nanoreactor exhibited prominent anti-cancer efficacy with minimal systemic toxicity.

**Conclusion:**

In summary, DHA@MIL-101 nanoreactor boosts CDT and ferroptosis for synergistic cancer therapy by molecular amplifier DHA. This work provides a novel and effective approach for synergistic CDT-ferroptosis with Chinese herbal monomer-DHA and Nanomedicine.

**Graphical Abstract:**

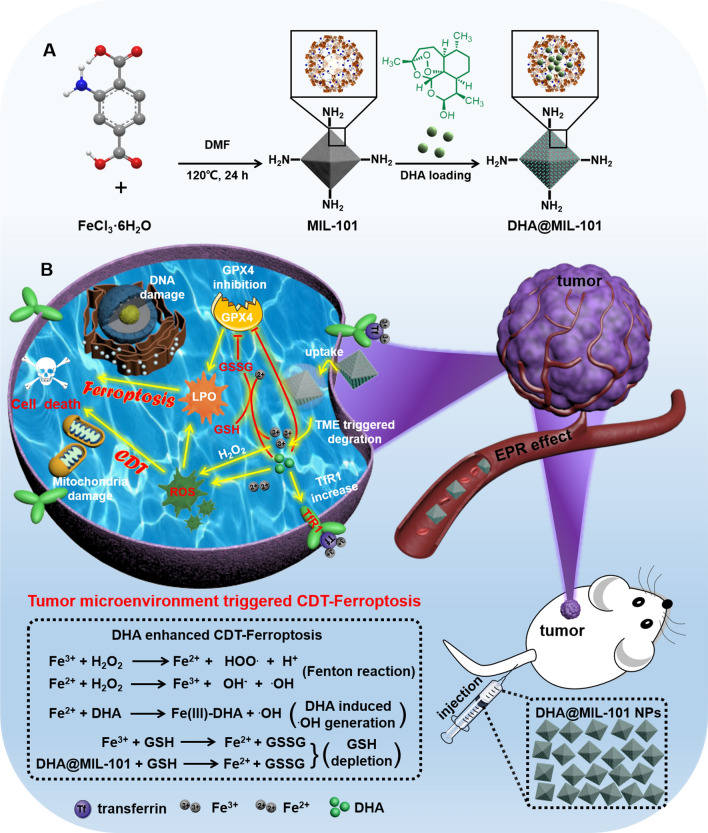

**Supplementary Information:**

The online version contains supplementary material available at 10.1186/s12951-022-01455-0.

## Introduction

Lung cancer ranks among the top three malignancies in terms of morbidity and mortality [1]. Because tumor cells are difficult to be completely removed by deep infiltration, resulting in a high risk of recurrence [2, 3]. Supplemental chemotherapy is therefore often required. Conventional chemotherapeutic agents are less selective in the treatment of lung cancer, leading to serious collateral damage to normal tissues [4–6]. Chemodynamic therapy (CDT) is a novel therapeutic strategy for the treatment of malignant tumor, which employs iron-involved Fenton or Fenton-like agents to produce highly deleterious reactive oxygen species (ROS) such as hydroxyl radical (**·**OH) in malignant cells and thereof induce cell apoptosis. The ROS generation by Fenton-type reactions requires neither oxygen (O_2_) nor an external light source, which enables CDT to avoid the major shortcomings of photodynamic therapy (PDT), such as limited laser penetration and hypoxia resistance in tumor tissues [7–11]. Despite its advantages including high tumor selectivity and low side effects, CDT technology remains in its infancy [12]. Recently, the main approach used in CDT is the low-valent transition metal ions can initiate Fenton-like reactions in cancer cells specifically to intracellular over-expressed hydrogen peroxide (H_2_O_2_) in the tumor microenvironment into highly reactive **·**OH, which induces oxidative stress and inactivates ambient cellular organelles and proteins instantaneously [13–16]. However, the insufficient amount of hydroxyl radicals limited the therapeutic effects due to the low level of H_2_O_2_ and transition metal ions concentration in the tumor microenvironment. Therefore, the development of an exogenous delivery of metal ions into tumor cells strategy to resolve this issue in CDT is highly desirable.

Of all the transition metal ions, iron ions (II)-dominated Fenton reaction has been developed as a typical nanocatalytic medicine for tumor-specific therapy. Notably, metal–organic framework (MOF), which are built by inorganic metal nodes (clusters or metal ions) with organic bridging ligands, has emerged as a promising class of functional materials that sparked increasing interest in the last few decades [17, 18]. Particularly, when scaled down to nanosize, nanoscale MOF can serve as unique materials platform for a variety of biomedical applications as facilitated by good biocompatibility, suitable size, delivery and controlled release of drug molecules [19]. Among these nMOF, iron-based MOF (e.g., MIL-53, MIL-88B, MIL-100, MIL-101, etc.) have attracted considerable attention due to their high porosity, such as large surface area, nontoxic nature, biodegradability and pH-responsive [20, 21]. The iron-based MOF can effectively release iron ions and further trigger the situ tumor-specific Fenton reaction to generate abundant toxic **·**OH under acidic tumor microenvironment, leading to efficient tumor inhibition. More importantly, **·**OH also can irreversibly peroxide the membrane lipids, causing more accumulation of lipid peroxide (LPO), which ultimately leads to ferroptosis, a type of iron-dependent nonapoptotic cell death [22]. Thus, different approaches have been explored to increase the concentration of iron ions in tumor cells [23]. However, the intratumoral H_2_O_2_ level is usually below the threshold H_2_O_2_ concentration (100 μM) because of the cellular redox homeostasis, which limits the therapeutic effect of **·**OH through Fenton reaction with iron ions [24]. The activity of nanoscale iron ion-based MOF alone is not sufficient to completely destroy the tumor thereupon. Iron-based MOF apoptosis-ferroptosis synergistic tumor therapy patterns are barely reported. To overcome this shortcoming, combined catalytic reactions are adopted that induce the concurrent generation of more ROS to enhance the anti-tumor efficacy.

In addition to iron ions-catalyzed ROS generation from H_2_O_2_, the peroxo compounds (ROOR) containing peroxide groups are also feasible for Fenton chemistry-mediated ROS-generating. According to previous literature reports, the ROOR molecules also can combine with iron ions (II) to form Fenton’s reagent, which has been extensively applied for highly efficient production of **·**OH [25]. Notably, dihydroartemisinin (DHA) is a sesquiterpene lactone compound extracted from Artemisia annua, family Asteraceae, which is recommended by the World Health Organization as a first-line antimalarial drug [26–28]. The cytotoxicity of DHA can be enhanced because its endoperoxide bridge be cleaved by iron ions to generate **·**OH [29, 30]. Accordingly, previous studies have revealed DHA can synergize with intracellular iron ions for tumor cell destruction [31–33]. But in healthy cells, the treatment with DHA is carried out without concern for its toxic effects as the iron ion content is extremely low [34–36]. However, DHA has poor water solubility and limited targeted enrichment in lung cancer tissues, restricting its anti-lung cancer application [37–39]. Therefore, we attempt iron-based MOF nanocarriers for delivery of DHA to improve **·**OH generation.

**Scheme 1:**
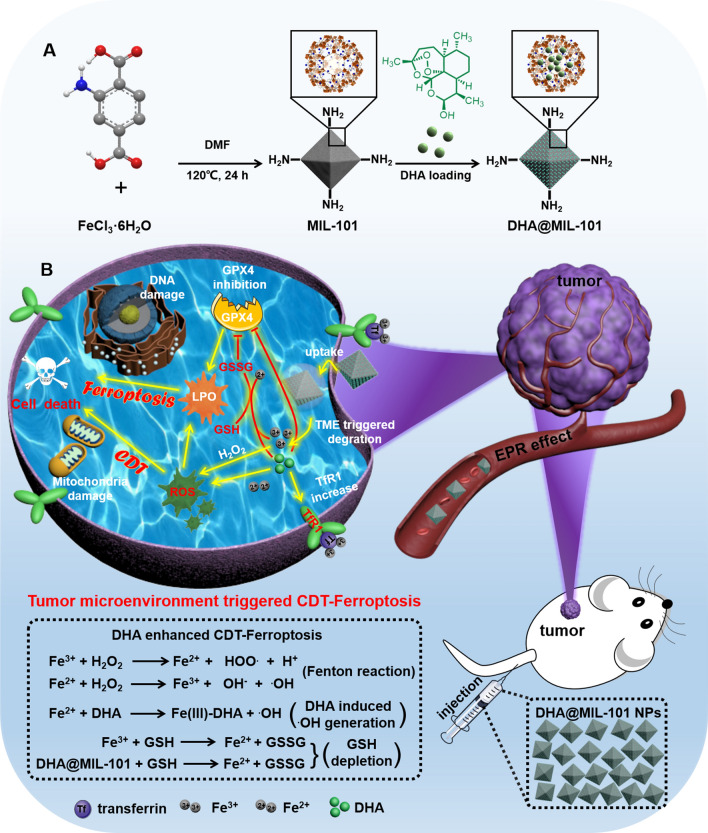
**A** Preparation procedure of DHA@MIL-101 NRs. **B** Schematic mechanism of DHA@MIL-101 NRs for synergistic CDT-ferroptosis therapy

Herein, we loaded DHA into the pores of peroxidase-like nanoscale metal–organic framework (nMOFs) MIL-101, to design activatable DHA@MIL-101 nanoreactor (NRs) for initiating Fenton chemistry-based CDT, whose application to construct pH/DHA cascade reaction for enhanced tumor treatment (Scheme 1A). As-constructed DHA@MIL-101 NRs were expected to possess the following favorable properties: (1) the MIL-101 NRs could simultaneously serve as the carrier of DHA molecules and peroxidase-like nanozyme, (2) the water solubility and biocompatibility of DHA were significantly improved after loading into MIL-101 NRs. (3) This device could degrade under the acid tumor microenvironment and thereby release DHA and iron ions. The DHA@MIL-101 NRs were internalized by cancer cells, and the degradation of DHA@MIL-101 NRs resulted in releasing of iron ions that could trigger **·**OH generation by Fenton reaction for CDT with the presence of endogenous H_2_O_2_. Furthermore, released DHA with peroxyl bridges generated **·**OH accompanied with iron ions [40]. DHA molecules from DHA@MIL-101 NRs not only served as an **·**OH amplifier consuming iron ions but also acted as a recruiter introducing iron ions influx by up-regulating transferrin receptor1 (TfR1) to generate increased ROS. On the other hand, ROS depleted glutathione (GSH) and inactivated glutathione peroxidase 4 (GPX4), leading to LPO accumulation to facilitate ferroptosis. Additionally, DHA molecules also acted as an efficient ferroptosis molecular amplifier by direct inhibiting GPX4. Finally, the excessive ROS and LPO damaged DNA and mitochondria, inducing cell apoptosis and exhibiting excellent anti-cancer efficacy thereupon (Scheme 1B). The current study performed in vitro and in vivo experiments to confirm the above conclusions, which provided a novel and efficient synergistic CDT-ferroptosis strategy for precise tumor therapy with Chinese herbal monomer-DHA and MOF.

## Results and discussions

### Characterization and microenvironmental response properties of DHA@MIL-101 NRs

**Fig. 1:**
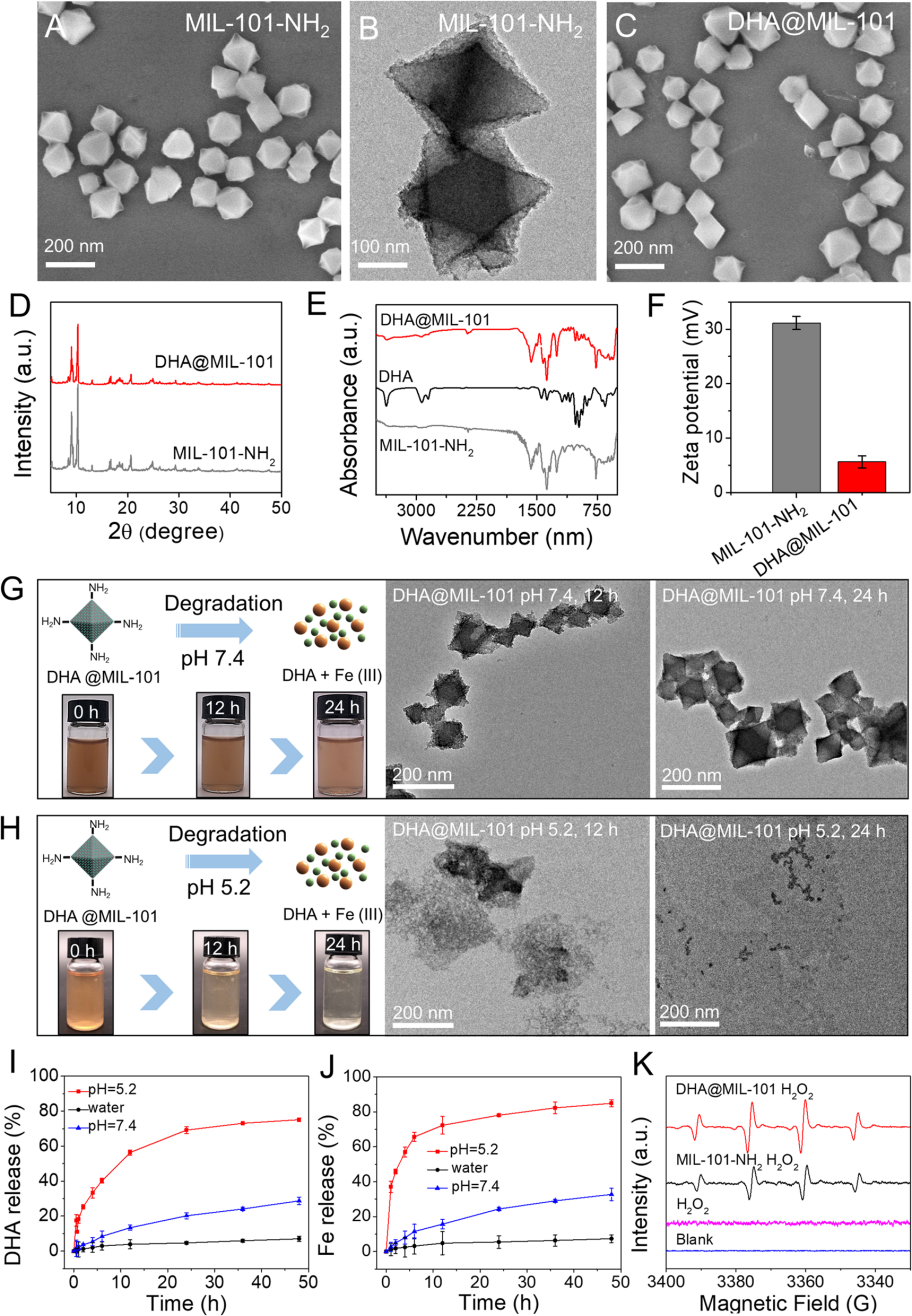
Characterizations and microenvironmental reactivity of DHA@MIL-101 NRs. **A**, **B** SEM and TEM images of MIL-101 NRs. **C** SEM images of DHA@MIL-101 NRs. **D** Powder XRD diffraction patterns of as-prepared MIL-101 NRs and DHA@MIL-101 NRs. **E** FTIR spectra of MIL-101 NRs, DHA and DHA@MIL-101 NRs. **F** Zeta potentials of MIL-101 NRs and DHA@MIL-101 NRs. **G**, **H** Schematic illustration, photos and morphology of DHA@MIL-101 NRs in response to pH (in PBS solutions with different conditions): **G** pH 7.4, treatment time is 12 h and 24 h, **H** pH 7.4, treatment time is 12 h and 24 h. **I** DHA molecules release profiles at the water, pH 7.4 and 5.2 from DHA@MIL-101 NRs. **J** iron ions release profiles at the water, pH 7.4 and 5.2 from DHA@MIL-101 NRs. **K** ESR analysis of **·**OH production of DHA@MIL-101 NRs at different systems by using DMPO as the spin-trapping agent

The preparation process of DHA@MIL-101 NRs were displayed in Scheme 1. MIL-101 was a typical isoreticular MOF built up from trimeric secondary building units (SBUs) with MIL-101 topology and has been reported as a nontoxic nanocarrier for the controlled delivery of several antitumoral and retroviral drugs [41]. MIL-101, [Fe_3_O(CH_3_COO)_6_] Cl was coordinated by 2-aminoterephtalic acid (NH_2_-BDC) ligands with Fe_3_O cluster were initially synthesized by a solvothermal method and possesses mesoporous channels of 29–34 Å [42]. The morphology and size of the as-synthesized, as well as the DHA-loaded MIL-101-NH_2_ nanoparticles (NPs), were characterized by SEM and TEM. MIL-101 NRs showed an almost identical size with an average size of 150 nm in diameter (Fig. 1A–C, Additional file 1: Fig. S1a). The hydrodynamic size of MIL-101-NH2 was measured to be about 246 nm. After loading the DHA, the average size of DHA@MIL-101 was determined to be 253 nm, similar to that of MIL-101-NH2 (Additional file 1: Fig. S1b). As shown in Fig. 1D, the crystallinity of the as-synthesized as well as the DHA@MIL-101 NRs were confirmed by their powder X-ray diffraction (PXRD) patterns, which matched well with the MIL-101 pattern. These results demonstrated that the structural integrity of MIL-101 remains unaltered when loaded with drugs. The Fourier transform infrared (FTIR) spectrum of DHA@MIL-101 NRs reveals some new absorption peaks in the range of 900–1200 cm^−1^ compared with the spectrum of free MIL-101, which can be assigned to the stretching vibration peak of O–O–C (peroxide) from pure DHA molecules (Fig. 1E). The effective encapsulation of DHA molecules in MIL-101 NRs was further confirmed by the featured ultraviolet−visible (UV–vis) spectrum (Additional file 1: Fig. S2). Additionally, as shown in Fig. 1F, the gradual decrease in the zeta potential of the MIL-101 NRs, also indicated the loading of DHA molecules. The DHA molecules’ loading content was quantified by thermal gravimetric analysis (TGA) under a nitrogen gas flow (see Additional file 1: Fig. S3). The DHA@MIL-101 NRs showed a large decrease of weight-loss at 180–650 ℃, and approximately 18 wt% weight-loss is observed, which originates from the removal of DHA molecules from DHA@MIL-101 NRs. UV–vis absorption analysis showed that the drug loading capacity efficiency of DHA molecules was 20 wt%, which was consistent with the value obtained from TGA.

Next, we explored the pH-responsive drug release of DHA@MIL-101 NRs under different mimic acidic tumor microenvironments. As shown in Fig. 1G, TEM images clearly showed that the morphology of DHA@MIL-101 NRs remained the same, and exhibited no obvious collapse under neutral conditions (pH = 7.4). Impressively, the hydrodynamic diameters of DHA@MIL-101 underwent little change during 48 h (Additional file 1: Fig. S1c), suggesting the excellent colloidal stability of DHA@MIL-101 NRs. In contrast, DHA@MIL-101 NRs degraded completely under an acidic condition (pH = 5.2) within 24 h incubation (Fig. 1H). An ideal pH-responsive drug delivery system must be stable under normal physiological conditions and biodegradable in slightly acidic tumor microenvironments. Therefore, the pH-responsive release of DHA molecules from DHA@MIL-101 NRs was also studied. The results showed that DHA@MIL-101 NRs exhibited a faster release of cargo drug in acidic conditions (pH 5.2) than in neutral conditions (pH 7.4). As shown in Fig. 1I, only 28.8% DHA molecules were released from the drug carrier after immersion in PH 7.4 conditions for 48 h. However, 75.6% DHA molecules were released after immersion in pH 5.2 conditions over an identical period time, which may be associated with the degradation of the MIL-101 frameworks. In addition, the iron ions release as a result of pH-responsive DHA@MIL-101 NRs degradation was investigated using ICP-MS. When the pH was down to 5.2, the iron ions release ratio was much fast than at pH 7.4, where an ultimate release ratio of 20% at pH 7.4 and 85% at pH 5.2 were recorded under the same condition for 48 h (Fig. 1J). The pH-responsive release feature of the MIL-101 NRs can allow the use of DHA@MIL-101 NRs in the clinic, demonstrating a controlled drug and iron-based Fenton catalyst agent delivery behavior for enhanced cytotoxicity toward tumor cells while lessening normal tissue toxicity. According to the above results, the pH-responsive specificity of DHA@MIL-101 NRs would benefit from the following aspects of promotion: (1) DHA@MIL-101 NRs could effectively accumulate at the tumor site through the permeability and retention effect (EPR) because of the suitable size. (2) The high H_2_O_2_ level of the tumor microenvironment would activate the DHA@MIL-101 NRs to switch on its CDT properties. (3) The DHA molecules release was further accelerated by the acidic tumor microenvironment, resulting in the enhancement of CDT. The DHA-involved in mediating H_2_O_2_-activated and acid microenvironment-responsive-enhanced process could not only heighten the contrast between tumor and normal tissues to raise the CDT precision, but also reinforce the anti-tumor therapeutic effect with relatively minimal collateral damages.

As is well-known, **·**OH radicals were the most toxic ROS, which could be generated by the Fenton reaction between Fe^3+^ and H_2_O_2_ involved Fenton agents. [43] To ascertain the **·**OH-generating ability of the DHA@MIL-101 NRs in the presence of H_2_O_2_, a trapping probe molecule 1,3-diphenylisobenzofuran (DPBF) was used to monitor the **·**OH generation by electron spin resonance (ESR) measurements. [44] As displayed in Fig. 1K, the MIL-101 group induced the generation of fourline spectrum of characteristic spectrum of DMPO/•OH with relative intensities of 1:2:2:1 compared with H_2_O_2_ and blank groups, demonstrating the capability of generating **·**OH in the presence of iron ions and H_2_O_2_. After DHA@MIL-101 NRs were introduced, the ESR signals showed the more **·**OH than that of MIL-101 NRs. Therefore, the ESR measurements confirmed that the NRs could cascade amplification **·**OH in the presence of DHA molecules. In all, these findings indicated acidic microenvironment promoted degradation of DHA@MIL-101 NRs, generating increased ROS by releasing cargo DHA molecules and iron ions thereupon.

### DHA@MIL-101 displayed enhanced cytotoxicity by boosting CDT-ferroptosis

After verifying the microenvironmental response performance of the DHA@MIL-101 NRs under given conditions. We next examined whether DHA@MIL-101 NRs could accumulate in cancer cells, then inhibit the cell viability. The cell internalization was evaluated using Lewis cells with Indocyanine-labeled DHA@MIL-101 (ICG-DHA@MIL-101) by flow cytometry and confocal microscopy. As shown in Additional file 1: Fig. S6, ICG-DHA@MIL-101 NRs-treated Lewis lung cancer cells (LLC) exhibited strong yellow fluorescence emission from ICG. Flow cytometry analysis also verified that Lewis cells treated with ICG-DHA@MIL-101 NRs exhibit higher fluorescence intensity than other treatments (Additional file 1: Fig. S6), consistent with the above observation. These results validated the effective cellular uptake of DHA@MIL-101 NRs on Lewis lung cancer cells, which guaranteed that the CDT and ferroptosis of DHA@MIL-101 NRs could be performed efficiently.

**Fig. 2:**
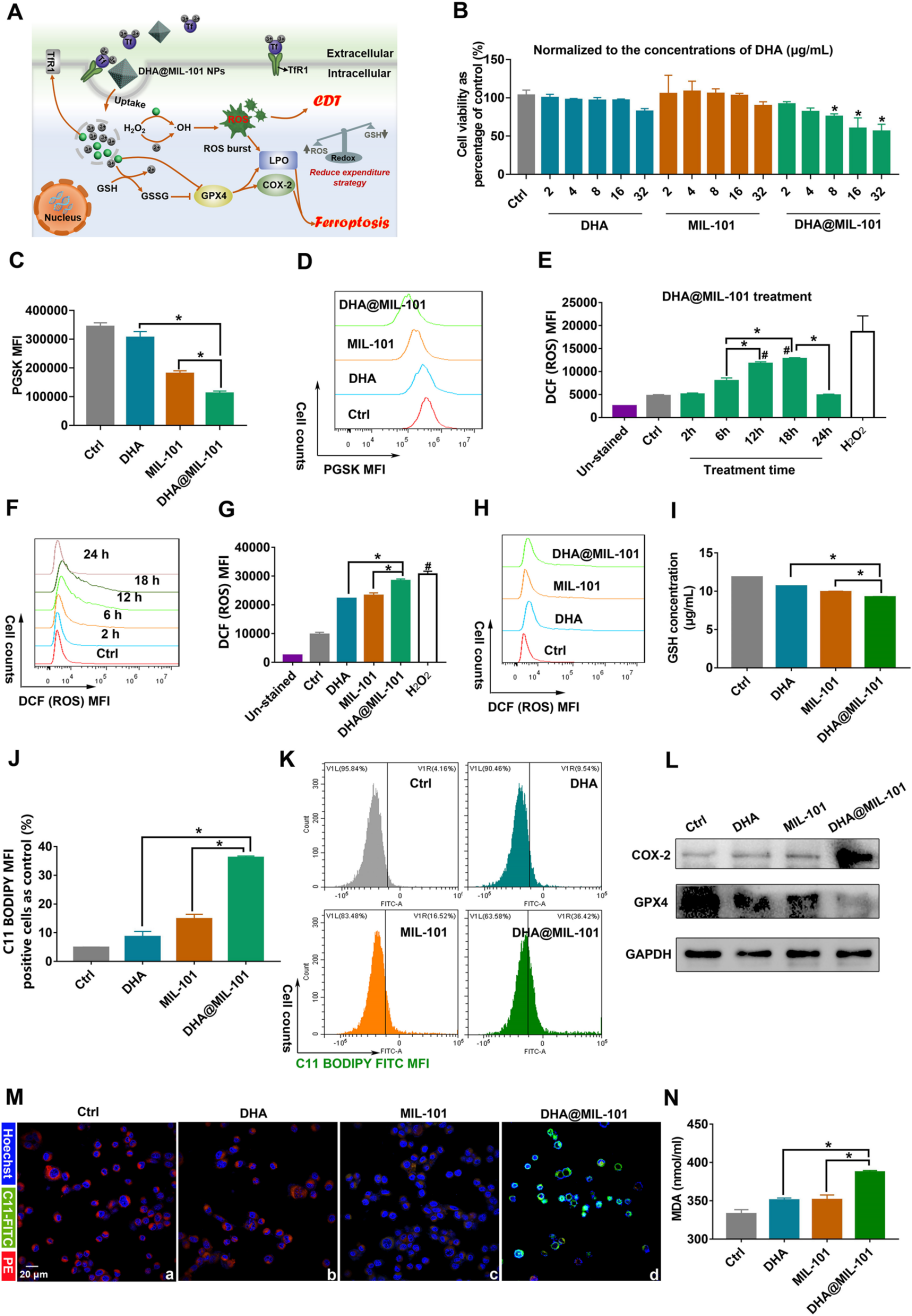
DHA@MIL-101 NRs treated Lewis cells exhibited enhanced cytotoxicity boosted by ROS generation and ferroptosis. **A** Schematic illustration of DHA@MIL-101 NRs mediated CDT and ferroptosis. **B** Different concentrations of DHA, MIL-101 NRs, and DHA@MIL-101 NRs were used to treat Lewis cells. Cell viability was analyzed by CCK-8. **C**, **D** Lewis cells were incubated with the DHA, MIL-101 NRs and DHA@MIL-101 NRs for 12 h and intracellular iron ions were detected using a PGSK probe and flow cytometry. **E**–**F** Lewis cells were incubated with DHA@MIL-101 NRs at various times to screen for the optimal time for inducing the peak of ROS. Cellular ROS generation was measured by DCFH-DA staining and flow cytometry. H_2_O_2_ treatment was a positive control. **G**, **H**) Lewis cells were incubated with DHA, MIL-101 and DHA@MIL-101 NRs for 18 h. Intracellular ROS was detected by DCFH-DA staining. **I** GSH content in Lewis cells was measured by a GSH kit. **J**, **K**, **M** Lipid peroxidation was detected using a C11-BODIPY probe with flow cytometry and confocal microscopy. **L** The expression of GPX4 and COX-2 in Lewis cells was measured by WB. **N** The MDA concentration in LLC was measured. All values are means ± SD (*n* = 3, * *p* < 0.05)

To demonstrate synergistic CDT and ferroptosis-induced cancer cell damage of DHA@MIL-101 NRs, in vitro cytotoxicity was then investigated by the cell-counting-kit-8 (CCK-8) assay. As shown in Fig. 2B, cell viability was thus significantly inhibited when the concentration of DHA@MIL-101 NRs up to 8 μg/mL (DHA concentration), while was not suppressed with DHA or MIL-101 NRs treatment. As expected, the incorporation of CDT and ferroptosis contributed to the highest cytotoxicity in the DHA@MIL-101 NRs.

As depicted in Fig. 1J, DHA@MIL-101 NRs possessed a pH-responsive controlled release capability, which should facilitate cargo DHA and iron ions release in the acidic tumor microenvironment. Further, the cellular iron ions were assessed by the PGSK probe. As shown in Fig. 2C, D, compared to cells treated with DHA or MIL-101 NRs, DHA@MIL-101 NRs treated-cells exhibited the weakest PGSK fluorescence after 12 h, indicating the highest increase in intracellular iron irons. Notably, the intracellular iron ions in DHA-treated cells also increased, which resulted from up-regulating TfR1 by the DHA molecule (Additional file 1: Fig. S7). As mentioned before, DHA molecules can synergize with iron ions for tumor cell destruction due to its unstable endoperoxide bridge (–O–O–) can be activated by iron ions to generate **·**OH. To detect the intracellular ·OH generation, a 2, 7-dichlorofluorescein diacetate (DCFH-DA) probe was used. As expected, DHA@MIL-101 NRs-treated cancer cells showed the strongest green fluorescence, revealing the highest **·**OH production in the DHA@MIL-101 NRs group, which was contributed to MIL-101 NRs sourced iron ions by Fenton reaction and the **·**OH amplifier DHA molecules (Fig. 2E–H). The above findings further demonstrated that DHA@MIL-101 NRs not only served as an **·**OH amplifier consuming iron ions but also acted as a recruiter introducing iron ions influx to generate increased ROS (Fig. 2A).

Subsequently, the concentration of GSH in Lewis cells incubated with DHA@MIL-101 NRs was measured by GSH assay kits. As displayed in Fig. 2I, after incubation with DHA@MIL-101 NRs, the content of intracellular GSH decreased by 22.1%, which was derived from the GSHOx-mimicking activities of DHA@MIL-101 NRs. Previous studies reported that the depletion of GSH could lead to glutathione peroxidase 4 (GPX4) inactivation, and then resulted in lipid peroxidation (LPO) accumulation to induce ferroptosis. Next, the level of intracellular GPX4 was investigated by western blot (WB). Compared with the control group, the treatment of MIL-101 NRs decreased GPX4 expressions, demonstrating the inactivation of a GPX4 signaling pathway. Additionally, the expression of GPX4 in the DHA treated group was also down-regulated slightly, probably based on its direct effect on protein, which was consistent with our previous findings [45]. Besides, the inactivating role of DHA@MIL-101 NRs was further promoted, owing to the combined effect of MIL-101 NRs and DHA molecules. Moreover, owing to the inactivation of GPX4, the obvious up-regulation of lipid peroxidation (LPO) was observed in DHA@MIL-101 NRs treated cells by Liperfluo probe (Fig. 2J–K, M). Quantitative flow cytometry analysis also verified that LPO induced by DHA@MIL-101 NRs is enhanced 4.5-fold and 2.4-fold respectively as compared to that MIL-101 NRs and DHA. Furthermore, the remarkable protein of ferroptosis, COX-2, up-regulated, accompanied by the production of LPO (Fig. 2J–K, M and Additional file 1: Fig. S16). Moreover, the product of ferroptosis, malondialdehyde (MDA), was also abnormally abundant in the DHA@MIL-101-treated LLC (Fig. 2N). Taken together, the above findings suggested DHA@MIL-101 NRs not only acted as an excellent **·**OH amplifier and iron ions recruiter to produce increased ROS to deplete GSH and inactivate GPX4, but also served as an efficient ferroptosis molecular amplifier by directly inactivating GPX4 to facilitate CDT and ferroptosis (Fig. 2A).

### DHA@MIL-101 NRs induced DNA and mitochondria damage

**Fig. 3:**
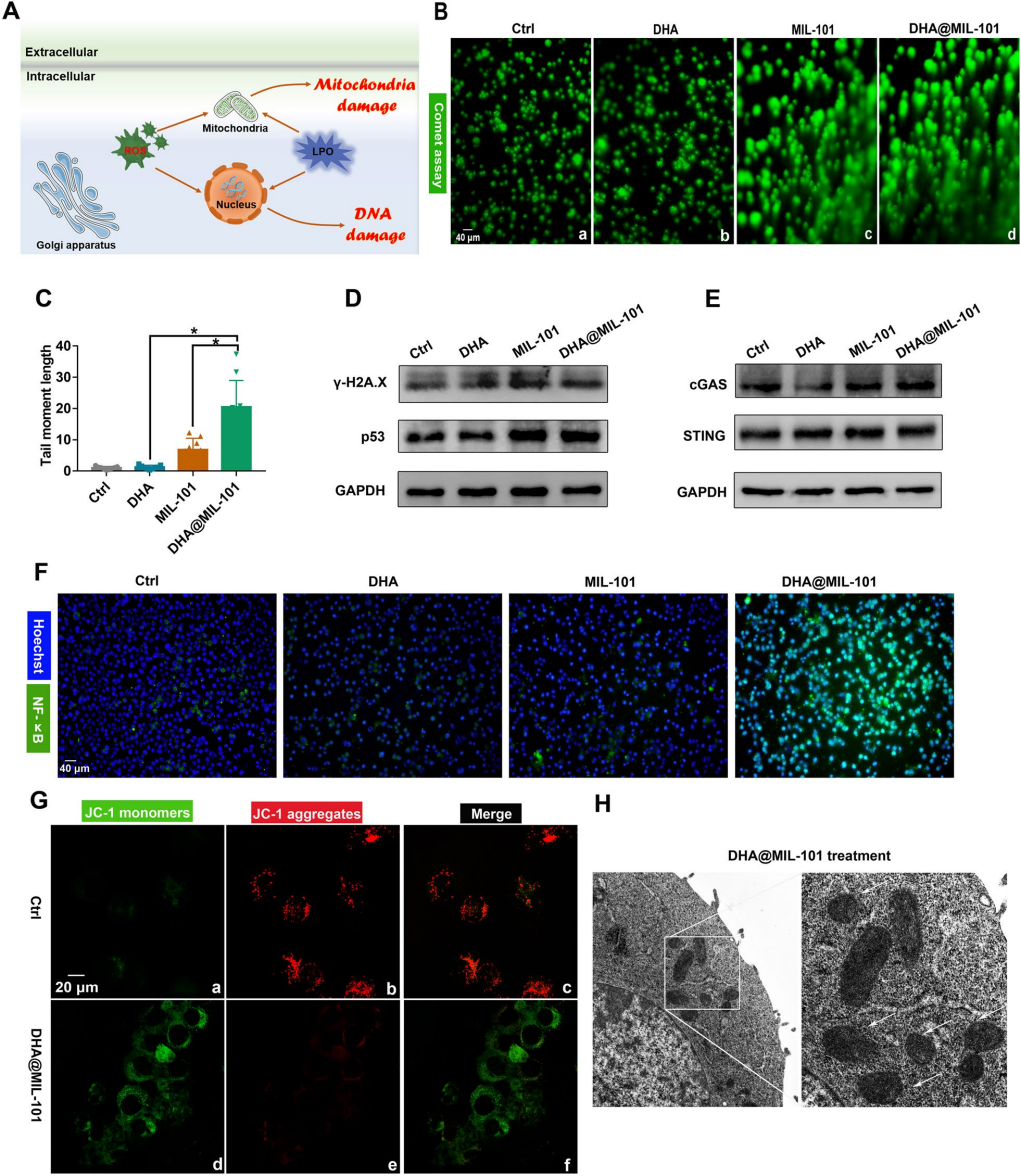
DHA@MIL-101 NRs facilitated DNA and mitochondria damage of Lewis cells. **A** Schematic illustration of DNA and mitochondria damage by DHA@MIL-101 NRs sourced ROS and LPO. **B**, **C** Lewis cells were incubated with DHA, MIL-101 NRs and DHA@MIL-101 NRs respectively. Comet experiments were applied to detect the DNA double-strand breaks (DDSB). The tail moment length was analyzed quantitatively via randomly selected cells. **D** The expression of p53 and γ-H2A.X, which are biomarkers of DNA damage, was assayed by WB. **E** The expression of cGAS, STING, which indicates DNA damage response (DDR), was assayed by WB. **F** The nuclear-translocation of NF-κB, which is direct evidence of NF-κB activation, was assayed by immunofluorescent staining. **G** Mitochondrial membrane potential was detected using a JC-1 probe with confocal microscopy. **H** The morphology of mitochondria was observed with TEM. All values are means ± SD (*n* = 3, * *p* < 0.05)

As is known, LPO can be formed by free radical attacks, such as ROS and reactive nitrogen species (RNS), on polyunsaturated fatty acid residues of phospholipids [46]. Excess LPO affects the metabolism and transport of substances, which in turn destabilizes the genome, leading to DNA damage [47]. What’s more, ROS can directly cause oxidative damage to genome DNA as well [48]. Therefore, we followed with an examination of the DNA damage in cancer cells treated with DHA@MIL-101 NRs (Fig. 3A). Firstly, the comet experiment was conducted to demonstrate the presence of DNA double-strand breaks (DDSB) in Lewis cells treated with DHA@MIL-101 NRs. As a result, obvious comet trails could be visualized, indicating DHA@MIL-101 NRs induced prominent DDSB (Fig. 3B, C). Notably, DHA treatment produced excess ROS and LPO but was much lower than the DHA@MIL-101 group. In general, serious DNA damage can not be repaired, leading to apoptosis eventually. Nevertheless, minor DNA damage can often be repaired in time so that DDSB does not occur in DHA-treated LLC. Furthermore, γ-H2A.X and p53, which can be activated after DNA damage, were found to up-regulate in the presence of DHA@MIL-101 NRs (Fig. 3D). Additionally, as shown in Fig. 3E, along with DDSB, the activation of cGAS and STING, is reported to be activated by fragmentation of DNA damage and subsequently promote nuclear translocation and phosphorylation of NF-κB, which was found to up-regulate in DHA@MIL-101 NRs-treated cells. Indeed, the activation of NF-κB was thus proved (Fig. 3F and Additional file 1: Figs. S8, S9). These findings strongly recommended that DHA@MIL-101 NRs triggered ferroptosis, resulting in DNA damage to cancer cells. In addition to attacking DNA, ROS and LPO are able to damage critical organelles e.g. mitochondria, which are the essential sites for cellular energy conversion [49] (Fig. 3A). The mitochondrial membrane potential of Lewis cells with JC-1 dye staining was detected next thereupon. As shown in Fig. 3G, strong green fluorescence was observed in DHA@MIL-101 NRs-treated cells, confirming apparent damage to mitochondria. Actually, mitochondrial shrinkage, the main characteristic of ferroptosis, was clearly observed by bio-TEM in Lewis cells treated by DHA@MIL-101 NRs, further proving the occurrence of mitochondria damage and ferroptosis (Fig. 3H). Altogether, DHA@MIL-101 NRs-mediated high-efficiency CDT and ferroptosis in tumor cells brought by ROS and LPO, further causing DNA and mitochondria damage thereupon (Fig. 3A).

### DHA@MIL-101 NRs possessed selective anti-lung cancer effect in vitro

**Fig. 4:**
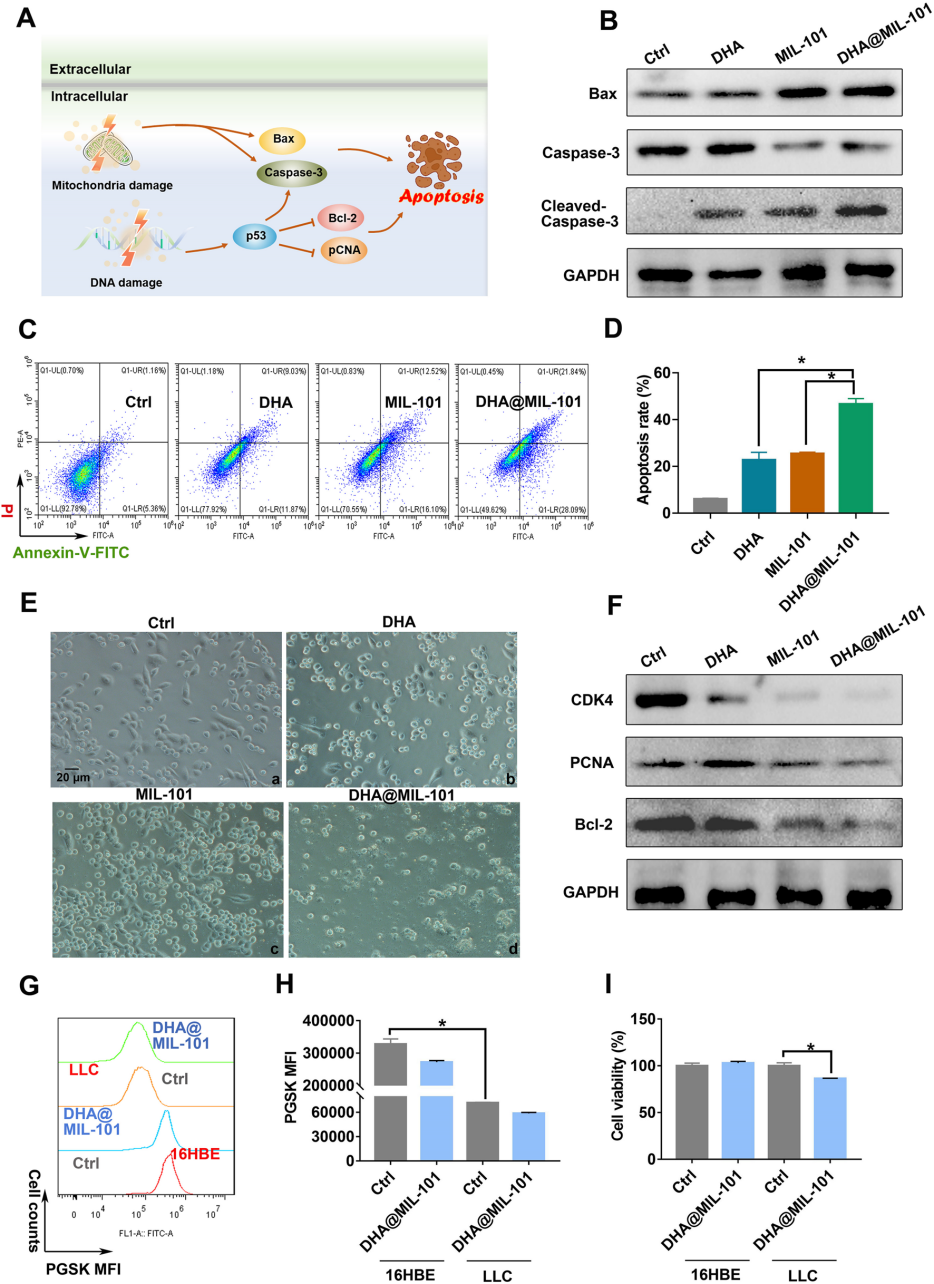
DHA@MIL-101 NRs exhibited a significant anti-lung cancer effect in vitro. **A** Schematic illustration of how DHA@MIL-101 NRs led to apoptosis of Lewis cells. **B** Lewis cells were treated with DHA, MIL-101 NRs and DHA@MIL-101 NRs for 24 h. The expression of apoptosis proteins Bax, caspase-3 was measured using WB. **C**, **D** The apoptosis rate was analyzed using Annexin-V/PI double staining and flow cytometry. **E** The cells were imaged with microscopy. **F** The expression of proliferation, anti-apoptosis proteins CDK4, PCNA and Bcl-2 were measured using WB. **G**, **H** DHA@MIL-101 NRs were utilized to treat 16HBE and LLC. Cellular iron ions were investigated by a PGSK probe. Decreased FITC fluorescence reflected accumulated iron ions. **I** The viability of 16HBE and Lewis cells was detected using CCK-8. Values are means ± SD (n = 3, * p < 0.05)

Loss of mitochondrial membrane potential leads to impaired cellular energy conversion, and aerobic phosphorylation, followed by activation of Bax and caspase-3, resulting in apoptosis in severe cases [50]. Moreover, when DNA damage is more severe, such as a strong DDSB, it cannot be repaired, at which time enhanced p53, Bax and caspase-3 expression is elevated to induce apoptosis [51]. Therefore, based on the previous findings, we speculate that DHA and iron ions synergize with each other to boost ferroptosis, producing LPO and thereby damaging DNA and mitochondria, which may induce apoptosis, inhibit proliferation and thus exert anti-cancer efficacy (Fig. 4A). In order to validate our hypothesis, Lewis cells were treated with DHA@MIL-101 NRs for 24 h, then graphed with microscopy. The results showed DHA@MIL-101 NRs treatment decreased the number of cells in the same field of view, making cells round, suggesting that apoptosis may occur (Fig. 4E). To further demonstrate the apoptosis rate, Annexin-V/PI double staining was applied to incubate Lewis cells. As presented in Fig. 4C, D, cells that received DHA@MIL-101 NRs treatment showed obvious apoptosis compared with DHA or MIL-101 NRs treatment alone. Moreover, the expression of apoptosis proteins Bax, cleaved-caspase-3 increased, whereas the expression of proliferation proteins CDK4, PCNA and anti-apoptosis protein Bcl-2 were decreased (Fig. 4B, F). To explore the targeting anti-cancer ability of DHA@MIL-101, normal and cancerous cells were carried out to investigate the DHA@MIL-101’s selective cytotoxicity. As displayed in Fig. 4I, the cell viability of DHA@MIL-101-treated 16HBE cells (a kind of normal bronchial epithelial cell) and Lewis lung cancer cells (LLC) was analyzed. The results showed DHA treatment significantly decreased the viability of LLC, but had little effect on the viability of 16HBE, indicating the targeting anti-cancer ability of DHA@MIL-101. Further findings revealed high basal iron ion levels in LLC and low basal iron ion levels in 16HBE cells as presented in Fig. 4G, H, which was consistent with many reports wherein cancer cells have an iron-overloaded cellular microenvironment [34–36]. This iron-overloaded microenvironment facilitated strong ferroptosis with the treatment of DHA@MIL-101. Therefore, It is an ingenious design to achieve selective cytotoxicity based on the difference in iron ion content in malignant cells and normal cells. These results strongly spoke that DHA@MIL-101 NRs selectively promoted apoptosis and inhibited proliferation of cancer cells after DNA and mitochondria damage, improving the anti-cancer efficacy of DHA (Fig. 4A).

### DHA@MIL-101 NRs exhibited prominent therapeutic efficacy in tumor-bearing mice

Finally, to verify the in vivo relevance of the in vitro findings, DHA, MIL-101 NRs and DHA@MIL-101 NRs were intravenously administrated into Lewis lung cancer-bearing mice. As shown in Additional file 1: Fig. S10, the results of in vivo imaging, organ imaging and tissue sections showed intense fluorescence could be observed in the tumor grafts of mice treated by ICG-DHA@MIL-101, indicating DHA@MIL-101 NRs were detection-friendly and can infiltrate into the lung cancer tumor. Interestingly and surprisingly, a little fluorescence of the drug was observed in the kidneys, but almost no fluorescence of the drug was detected in the liver and spleen, indicating that the ICG-modified DHA@MIL-101 NRs were barely distributed in the liver and spleen after being injected intravenously. The neutral charge with excellent biocompatibility and water solubility of DHA@MIL-101 NRs display enhanced accumulation at the tumor sites and prolonged intratumor retention due to the enhanced permeability and retention (EPR) effect. Generally speaking, the 160-nm sized nanoparticles accumulated in the liver after intravenous administration preferentially, where converses the nanoparticles for excretion by the kidneys and intestines [52]. Nevertheless, the DHA@MIL-101 NRs with easy degradation could be eliminated by the liver faster and transferred into the kidneys compared with hard-nanomaterial particles. As shown in Fig. 1G, H, the easily-degraded feature of DHA@MIL-101 NRs had already been confirmed. This may be why no hepatic, spleen uptake after intravenous injection of DHA@MIL-101 NRs, but intense tumor and kidney uptake.

**Fig. 5:**
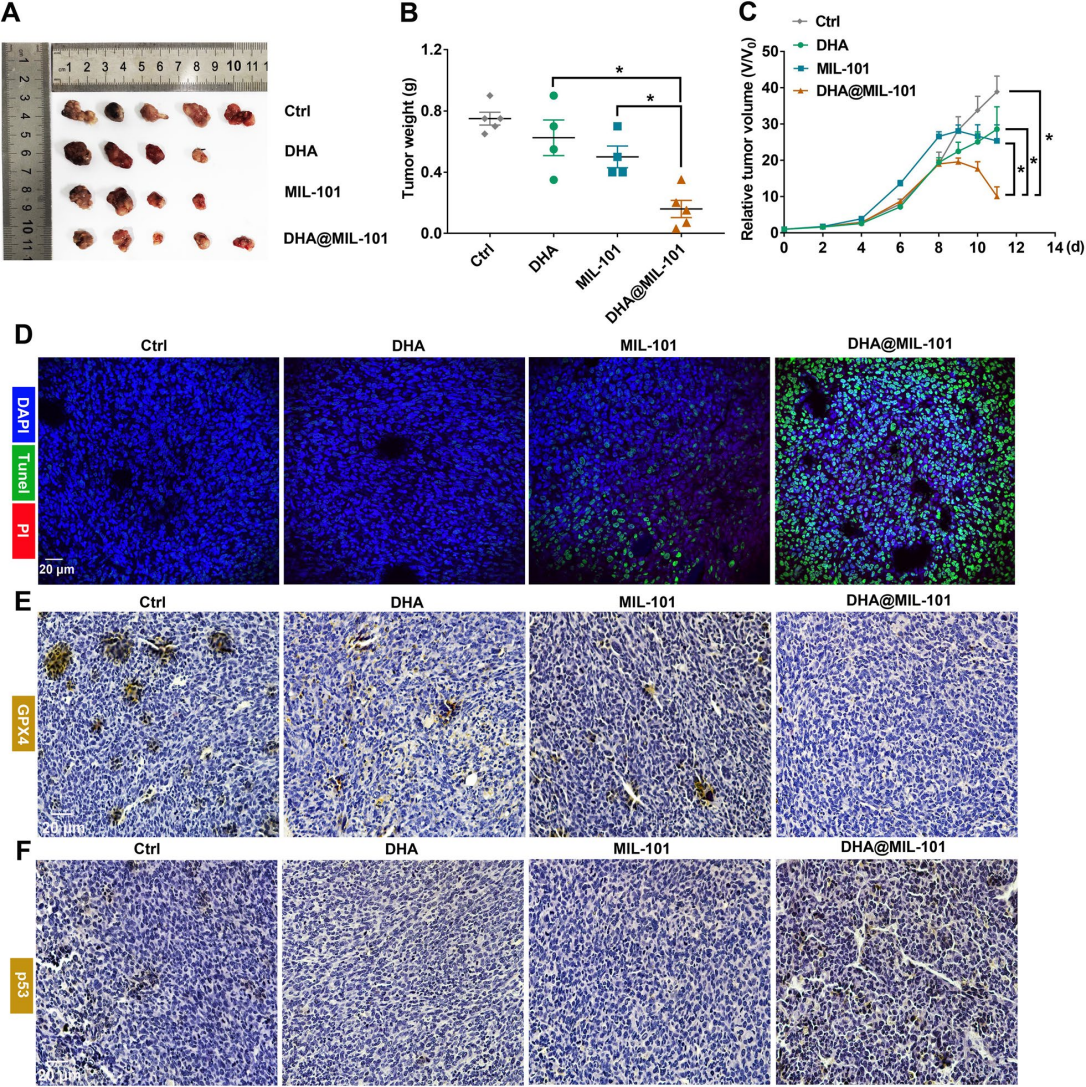
DHA@MIL-101 NRs exhibited significant efficacy in vivo and induced powerful apoptosis, ferroptosis, and DNA damage in tumor grafts. Caudal vein injection of DHA, MIL-101 NRs and DHA@MIL-101 NRs in 200 μL of PBS per mouse. **A** The mice were sacrificed and tumor grafts were harvested, then photographed. **B** The weight of tumor grafts was measured. **C** The tumor growth of Lewis cells-bearing mice was monitored during the whole experiment. **D** The tumor tissues were prepared for paraffin sections, then labeled with DAPI, Tunel and PI. The apoptosis and necrosis were assayed using confocal microscopy. **E**, **F** Expression of GPX4 (biomarkers of ferroptosis) and p53 (biomarkers of DNA damage) was detected with IHC staining. Values are means ± SD (*n* = 5, * *p* < 0.05)

In addition, to evaluate the ferroptosis, DNA damage and therapeutic efficacy of DHA, MIL-101 NRs and DHA@MIL-101 NRs were injected into Lewis lung cancer-bearing mice every day at the same dose of DHA or MIL-101 (5 mg/kg). After consecutive treatments, the volume change and weight of tumor grafts were counted. At the same time, tumor grafts were extracted for histological analysis. As displayed in Fig. 5, In the tumor grafts of DHA@MIL-101 NRs-treated mice, significant expression of p53 and decreased expression of GPX4 along with enhanced COX-2, γ-H2.AX expression, which indicated induction of ferroptosis and DNA damage, was observed (Fig. 5E, F and Additional file 1: Fig. S11). More importantly, as presented in Fig. 5A–C, DHA@MIL-101 NRs treatment obviously suppressed the tumor growth. Based on the in vitro results, we speculated that DHA molecules and iron ions released in tumor grafts may induce significant apoptosis of cancer cells. As expected, Tunel staining of tumor tissue sections showed prominent green fluorescence and PI signal in mice treated with DHA@MIL-101 NRs (Fig. 5D and Additional file 1: Fig. S12), which evidenced DHA-induced malignant cell apoptosis. Moreover, the expression of Bax and caspase-3, which are biomarkers of apoptosis, was increased in mice that received DHA@MIL-101 treatment. But the expression of proliferation protein PCNA decreased (Additional file 1: Fig. S13). In all, DHA@MIL-101 NRs exhibited increased therapeutic efficacy in inducing ferroptosis and DNA damage in Lewis lung cancer-bearing mice.

To study the side effects of DHA@MIL-101 NRs, the body weight and HE staining of vital organs were analyzed. As shown in Additional file 1: Fig. S14, all animals in the DHA@MIL-101 NRs groups, displayed a similar change in their body weight over the in vivo experiment duration, whose body weight curves overlap that of the control, DHA, MIL-101 NRs groups. HE staining also showed no apparent tissue damage to the heart, liver, spleen, lungs, and kidneys (Additional file 1: Fig. S15). In a word, the injected DHA@MIL-101 NRs and associated MOFs had virtually minimal side effects.

## Conclusions

In summary, DHA@MIL-101 NRs were successfully developed from MIL-101 NRs, then applied for anti-cancer CDT with the following favorable properties: (1) the MIL-101 NRs could simultaneously serve as the carrier of DHA molecules and peroxidase-like nanozyme. (2) the water solubility and biocompatibility of DHA molecules were significantly improved after loading into MIL-101 NRs. (3) This device could degrade under the acid tumor microenvironment and thereby release DHA molecules and Fe ion, production of increased ROS and triggering ferroptosis by the effect of molecular amplifier and motor. Ferroptosis is a novel pattern of cell death that regulates the immune microenvironment of malignant tumors and promotes immunotherapy [53], which is also an essential access point for our future research. In the current study, we highlight how DHA@MIL-101 NRs act as a straightforward anti-cancer therapeutic agent. For the first time, ROS and LPO produced by DHA@MIL-101 NRs were verified to damage cellular DNA and mitochondria, leading to final apoptosis thereupon. The DHA@MIL-101 NRs are ROS and LPO precursors, which could induce prominent oxidative damage to cancer cells’ DNA and mitochondria through interaction among DHA, iron ions and H_2_O_2_ as illustrated in Scheme 1. The present work provides a novel and efficient synergistic CDT-ferroptosis strategy for precise tumor therapy with Chinese herbal monomer-DHA and MOF, where the effect of increased free radical production and prominent ferroptosis induced by the DHA and Fe irons are elucidated as well.

## Methods and materials

### Preparation of DHA@MIL-101 NRs

MIL-101 nMOF was synthesized according to an approach described in the literature with some modifications. In short, 360 mg 2-aminoterephthalic acid and 1 g FeCl_3_·6H_2_O were dissolved in 40 mL DMF. After being treated at 120 ℃ for 20 h, the brown powder was centrifuged at 10,500 rpm for 20 min and washed three times in turn with DMF and ethanol, respectively. At last, the sediment was resuspended in ethanol and stored at 4 °C. The as-synthesized MIL-101 (10.0 mg) was dispersed in ethanol (5 mL). Then DHA (5.0 mg) dissolving in 2 mL ethanol was added to the dispersion of MIL-101 and magnetically stirred for 24 h in the dark at room temperature. Finally, the nanosuspension was washed with distilled water (500 μL × 5 times) to remove the free drug. Ultrasonic suspension of the nanosuspension for one minute each time distilled water was added for washing. The obtained DHA@MIL-101 NRs was stored at 4 ℃ prior to be used. All concentrations and dosages of DHA@MIL-101 NRs normalized to DHA.

### Preparation of ICG-DHA@MIL-101 NRs

30 mg DHA@MIL-101, EDC (100 mg, 0.52 mmol) and NHS (100 mg, 0.87 mmol) were dissolved in water (10 ml) and stirred for 2 h at room temperature to activate the carboxyl groups. ICG (5 mg, 6.5 μmol) was added into the solution under vigorous stirring for 24 h in the dark at room temperature and washed with water five times to obtain ICG-DHA@MIL-101 NRs. ICG- DHA@MIL-101 NRs was stored at 4 ℃ prior to be used. All concentrations and dosages of ICG-DHA@MIL-101 NRs normalized to DHA.

### Characterization of DHA@MIL-101 NRs and ICG-DHA@MIL-101 NRs

We collected all the water in washing procedures and the supernatant at the beginning to measure the loading content of the drug by the UV–vis absorption spectra technique. DHA could be converted into a UV absorbing compound through incubating with NaOH (2%) at 50 °C for 30 min and detected by the characteristic UV absorbance at 290 nm. The morphology and size of DHA@MIL-101 NRs were observed using scanning electron microscopy (SEM) and Transmission electron microscopy (TEM). To assay the hydrated size and zeta potential, the DHA@MIL-101 NRs was distributed in ultrapure water, and analyzed by Malvern laser particle size analyzer. Finally, the X-ray diffraction, infrared spectroscopy and thermal weight loss analysis (TGA) were applied to detect the successful preparation of DHA@MIL-101 NRs. Furthermore, the successful attachment of ICG to DHA@MIL-101-NH_2_ was detected through special absorption at 790 nm using UV–Vis absorption spectroscopy.

### Detecting free radical by ESR

The generation of free radical induced by Fe^2+^ and DHA was detected using 5,5-dimethyl-1-pyrroline N-oxide (DMPO) by ESR. Briefly, MIL-101 NRs (0.1 mL, 5.0 mg/mL) and DHA@MIL-101 NRs (same quality of MIL-101) solution at pH 5.2 in PBS, followed by incubation at 37 ℃ for 1 h. Then DMPO (0.05 mL, 0.5 M) was added as the spin–trapping agent, and the 1:2:2:1 multiplicity characteristic peak of DMPO-OH adducts were recorded by ESR immediately. As control, DMPO group was also tested for comparison.

### Analysis of PH-responsive DHA and ferric iron release

DHA@MIL-101 NRs (10 mg) were packaged into a dialysis bag (MWCO = 3500), then immersed within 20 mL of phosphate-citrate buffer (pH 5.2) and phosphate buffer solution (PBS, pH 7.4), respectively at 37 ℃ in a beaker. At different time points, 2.0 mL solution was collected to determine the concentration of DHA using UV–vis spectra, then 2.0 mL fresh PBS was added back to the beaker. After centrifugation, the released iron ions were analyzed by ICP-MS. Three independent experiments were carried out to minimize the deviations.

### Cell models and treatments

Lewis lung cancer cells (LLC) were used to as a lung cancer cell model. 16HBE cells were used as a kind of normal bronchial epithelial cell model. These cells were purchased from the Cell Bank of Shanghai Institutes for Biological Sciences (Shanghai, China). The cells were cultured in DMEM medium (Sigma-Aldrich, St Louis, USA) supplemented with 10% fetal bovine serum (QmSuero/Tsingmu Biotechnology, Wuhan) in a humidified incubator (5% CO_2_/95% air atmosphere, 37℃). In the in vitro experiments, DHA concentrations of DHA, DHA@MIL-101 NRs and ICG-DHA@MIL-101 NRs were all 8 μg/mL. The concentration of MIL-101 NRs was the same as the total concentration DHA@MIL-101 NRs.

### Drug uptake of ICG-DHA@MIL-101 NRs

Lewis cells were seeded onto 24-well plate or confocal dish with a density of 2 × 10^5^ cells, then incubated with DHA, MIL-101 NRs and ICG-DHA@MIL-101 NRs for 12 h. The cells were harvested for flow cytometry (flow cytometry) analysis (Cytoflex, Beckman Coulter, USA). The cells on confocal dish were fixed with paraformaldehyde, nuclear stained with Hoechst 33,342, then imaged using a laser scanning confocal microscopy (FV3000RS, Olympus, Japan).

### ROS detection

Lewis cells were plated in 24-well plates with a density of 2 × 10^5^ cells per well and incubated with DHA@MIL-101 NRs for various times (2, 6, 12, 18, 24 h). In addition, for compared analysis, different agent (DHA, MIL-101 and DHA@MIL-101) were also performed. After treatment, the cells were incubated with 10 μM of 2,7-Dichlorodi-hydrofluorescein diacetate (DCFH-DA, S0033, Beyotime, Shanghai, China) at 37℃ for 40 min before being harvested and assayed by flow cytometry. H_2_O_2_ treatment was applied as the positive control.

### Viability assay of cells

For assay of viability, Lewis cells or 16HBE cells were seeded onto 96-well plates with a density of 1 × 10^4^ cells per well and treated by DHA, MIL-101 NRs, DHA@MIL-101 NRs respectively for 24 h. Viability of cells in the 96-well plates were assayed using a CCK-8 kit (HY-K0301, MCE, NJ, USA). Briefly, the CCK-8 was incubated with cells for 2–4 h. The absorbance at 450 nm of cells in 96-well plates was measured using a Multifunctional Enzyme Labeler (SpectraMax i3, Molecular devices).

### Ferroptosis analyze of Lewis cells

For ferroptosis analyze of Lewis cells, the intracellular ferric irons, GSH content, expression of GPX4, COX-2, and lipid peroxidation were detected respectively. According to the manufacturer’s instruction, PGSK probe (GC40243, GLPBIO Technology Inc, USA) combined with flow cytometry was applied to detect the concentration of intracellular ferric irons. Alternatively, in light of the manufacturer’s instruction, the concentrations of intracellular GSH were measured by a biochemical assay kit (E-BC-K030-S, Elabscience). Furthermore, the expression of GPX4 and COX-2 was measured using Western blotting (WB). Finally, the production of lipid peroxidation was assayed by a C11-BODYPI probe (GC40165, GLPBIO Technology Inc, USA), whose fluorescence covert to FITC from PE indicates accumulation of lipid peroxidation in Lewis cells. Additionally, malondialdehyde (MDA) concentration in Lewis cells was measured by a MDA assay kit (E-BC-K025-S, Elabscience).

### DNA and mitochondria damage assay

Comet assay were performed to evaluate the DNA double strand break (DDSB). Lewis cells were seeded in 24-well plates, treated as required. Cell suspensions in PBS were prepared and mixed with low melting point agarose (LMPA). The mixture was then dripped onto a glass slide pre-coated with agarose gel and pressed with glass, followed by electrophoresis at 25 V, 250 mA for 25 min. The mixture was then lysed in alkaline lysis solution and neutralized using tris–Hcl (PH = 6.0). Hoechst 33,342 was applied to stain nuclei. Images were acquired by fluorescence microscopy. Alternatively, proteins exacted from Lewis cells were used to measure the expression of p53 and γ-H2A.X, which are biomarkers of DNA damage response. Finally, JC-1 probe (A3516, APExBIO Technology Inc, USA) was applied to analyze the membrane potential to confirm the degree of mitochondria damage. Alternatively, the cells treated with DHA@MIL-101 NRs was prepared prepared as ultrathin sections for TEM observation.

### Evaluation of in vitro anti-cancer efficacy

Lewis cells were treated by DHA, MIL-101 NRs and DHA@MIL-101 NRs for 24 h. Cell number and morphological change were observed by microscopy. In addition, the apoptosis rate of cells was assayed by FITC-Annexin-V/Propidium iodide (PI) double staining (Purchased from CHAMOT BIOTECHNOLOGY CO., LTD.) and flow cytometry. Alternatively, the expression of proliferation proteins (PCNA, CDK4 and Bcl-2), apoptosis proteins (Bax, cleaved-caspase-3) was measured using WB technique.

### Flow cytometry and Annexin-V/PI staining

Lewis cells was acquired on a Beckman Cytoflex flow cytometer. FITC-Annexin-V, DCFH-DA, PGSK, C11-BODYPI-FITC fluorescence was acquired in the FITC channel. PI fluorescence was acquired in the PE channel. ICG-DHA@MIL-101 NRs fluorescence was acquired in APC-A750 channel. At least 1 × 10^4^ cells/per sample were acquired. Geometric means (GM) were used to quantify the fluorescent intensity. For Annexin-V/PI assay, cells were incubated with Annexin-V-FITC and PI (Purchased from CHAMOT BIOTECHNOLOGY CO., LTD.) for 10 and 5 min respectively, then harvested on flow cytometry. The apoptosis of cells was assayed through calculate the Annexin-V positive cells.

### Western blotting (WB) measurement

Lewis cells treated as mentioned before in 6-well plates were lysed in RIPA buffer with protease inhibitor. Cell lysates were cleared by centrifugation and protein concentration determined using a BCA assay kit. Equal protein aliquots (10 μg) were separated by SDS-PAGE electrophoresis and transferred to a PVDF membranes. The membranes were blocked with 5% bovine serum albumin in TBST and then incubated with antibodies of PCNA (bs-2006R, Bioss, Beijing, China), Caspase-3 (19677-1-AP, Proteintech, Wuhan, China), Bax (50599-2-Ig, Proteintech, Wuhan, China), Bcl-2 (26593-1-AP, Proteintech, Wuhan, China), CDK4 (11026-1-AP, Proteintech, Wuhan, China), p53 (bs-2090R, Bioss, Beijing, China), γ-H2A.X (bs-3185R, Bioss, Beijing, China), GPX4 (14432-1-AP, Proteintech, Wuhan, China), COX-2 (A1253, Abclonal, Wuhan, China), and GADPH (PMK053C, BioPM, Wuhan, China) overnight at 4 °C. Horseradish peroxidase-conjugated secondary antibodies were used to bind to primary antibodies as mentioned above. Protein bands were imaged using a ECL luminescent liquid (PMK003, BioPM, Wuhan, China). The bands were exposed using a Bio Imaging system (170-8265, Bio-Rad).

### RT-PCR analysis

The mRNA was quantified to 1 μg, then reverse transcripted to cDNA using a transcriptor cDNA synthesis kit (PC5801, TRUEscript RT MasterMix, Beijing, Aidlab). Fluorescence real-time quantitative PCR was performed using an SYBRGreen real time PCR Master Mix kit (PC3301, Beijing, Aidlab). The Primers (5′ to 3′) sequences were as follows:

Mouse GAPDH Forward: AGGTCGGTGTGAACGGATTTG.

Mouse GAPDH Reverse: TGTAGACCATGTAGTTGAGGTCA.

Mouse TfR1 Forward: CTGGCTCTCACACTCTCTCAGCTTT.

Mouse TfR1 Reverse: GCATTTGCGACTCCCTGAATAGTCC.

### Lewis cells-bearing mouse model and treatments

Female C57 mice at 5–6 weeks of age (18 ~ 20 g) were purchased from Laboratory Animal Center at the Hubei University of Medicine (Hubei, China). Animal handling and experimental procedures were in line with protocols approved by the Animal Care Committee at the Hubei University of Medicine. Mice were housed in a temperature-controlled environment with fresh water and rodent diet available at all times. All inoculations and administrations were performed under Nembutal anesthesia. Each mouse was subcutaneously injected at the right haunch with Lewis cells (2 × 10^6^ cells/200 μL in PBS). The animals were randomly grouped (5 mice per group) when the tumor growth reached roughly 500 mm^3^. Injections of DHA, MIL-101 NRs and DHA@MIL-101 NRs, each in 200 μL of PBS per mouse, were administered through the caudal vein. For DHA and DHA@MIL-101 NRs, dosages were normalized to 5 mg/kg of DHA. The concentration of MIL-101 NRs was the same to MIL-101 in DHA@MIL-101 NRs. The fluorescence distribution of nanoagent was detected using in vivo imaging at 12 h after administration. The mice were treated with the various agent a total of 3 times, every 24 h. Finally, the mice were sacrificed and vital organs were harvested and imaged for drug fluorescence. Cryosections (5 μm) of tumor tissues and vital organs were prepared for fluorescent microscopy and immunohistochemical staining.

### HE, immunohistochemical, tunel and immunofluorescent staining

Briefly, paraffin sections were dewaxed, rehydrated, antigen repaired with sodium citrate for 20 min. For HE staining, the paraffin sections were stained by Eosin and hematoxylin. According to the manufacturer’s instruction, tunel and PI were used to label the apoptotic cells and necrotic cells firstly, then DAPI was applied to stain the nuclei. For IHC staining, the paraffin sections were then incubated in 3% hydrogen peroxide for 12 min at room temperature. The paraffin sections were blocked with 5% BSA for 40 min, stained with primary antibodies overnight at 4 ℃, then stained with secondary antibody (PV-9000, ZSGB-BIO, Beijing, China) for 1 h at 37 ℃. Diaminobenzidine (DAB, ZLI-9018, ZSGB-BIO, Beijing, China) was applied for coloration for 1–3 min at room temperature. Hematoxylin was used to stain the nucleus. Primary antibodies included PCNA (bs-2006R, Bioss, Beijing, China), Caspase-3 (19677-1-AP, Proteintech, Wuhan, China), Bax (50599-2-Ig, Proteintech, Wuhan, China), p53 (bs-2090R, Bioss, Beijing, China), γ-H2A.X (bs-3185R, Bioss, Beijing, China), GPX4 (14432-1-AP, Proteintech, Wuhan, China), COX-2 (A1253, Abclonal, Wuhan, China). For nanagent fluorescent microscopy, cryosections were dewaxed, rehydrated, then stained by DAPI for labeling of the nucleus.

### Statistical analysis

Statistical differences between groups were analyzed using One-way analysis of variance (ANOVA). Statistical significance was determined by Turkey Test. The p value less than 0.05 was considered to be statistically different.

## Supplementary Information

**Additional file 1: Figure S1.** Size distribution of MIL-101-NH2, hydrodynamic diameters and stability evaluation of DHA@MIL-101. **Figure S2.** UV–vis absorption spectra of DHA, MIL-101-NH2 and DHA@MIL-101-NH2. **Figure S3.** TGA curves of MIL-101-NH2 and DHA@MIL-101-NH2. **Figure S4.** SEM images of ICN-DHA@MIL-101. **Figure S5.** UV–vis absorption spectra of ICG and ICG-DHA@MIL-101-NH2. **Figure S6** Uptake of ICG-DHA@MIL-101 in LLC. **Figure S7.** DHA up-regulated the expression of TfR1 in LLC. **Figure S8.** DHA@MIL-101 promoted Nuclear translocation of NF-κB of LLC. **Figure S9.** DHA@MIL-101 promoted phosphorylation of NF-κB of LLC. **Figure S10.** The ICG-DHA@MIL-101 distribution in tumor grafts. **Figure S11.** The COX-2 and γ-H2A.X expression in tumor grafts. **Figure S12.** DHA@MIL-101 induced prominent apoptosis and necrosis of cancer cells in tumor grafts. **Figure S13.** DHA@MIL-101 promoted the expression of Bax, caspase-3, decreased expression of PCNA in tumor grafts. **Figure S14.** The body weight of LLC-bearing mice varied little after DHA@MIL-101 treatment. **Figure S15.** No evident organ toxicity in DHA@MIL-101-treated mice. **Figure S16.** LPO in LLC was detected using C11-BODIPY probe with confocal microscopy. **Figure S17.** Quantitative gray analysis of WB results in Fig. 2 to Fig. 4.

## Data Availability

All data generated or analyzed during this research included in this manuscript.
