# Supplementary material for: A nanoreactor boosts chemodynamic therapy and ferroptosis for synergistic cancer therapy using molecular amplifier dihydroartemisinin
Source: J Nanobiotechnology. 2022 May 14;20:230. doi: 10.1186/s12951-022-01455-0 (PMC9107746; doi:10.1186/s12951-022-01455-0)
Supplement: Supplementary file 1 — Additional file 1: Figure S1. Size distribution of MIL-101-NH2, hydrodynamic diameters and stability evaluation of DHA@MIL-101. Figure S2. UV–vis absorption spectra of DHA, MIL-101-NH2 and DHA@MIL-101-NH2. Figure S3. TGA curves of MIL-101-NH2 and DHA@MIL-101-NH2. Figure S4. SEM images of ICN-DHA@MIL-101. Figure S5. UV–vis absorption spectra of ICG and ICG-DHA@MIL-101-NH2. Figure S6 Uptake of ICG-DHA@MIL-101 in LLC. Figure S7. DHA up-regulated the expression of TfR1 in LLC. Figure S8. DHA@MIL-101 promoted Nuclear translocation of NF-κB of LLC. Figure S9. DHA@MIL-101 promoted phosphorylation of NF-κB of LLC. Figure S10. The ICG-DHA@MIL-101 distribution in tumor grafts. Figure S11. The COX-2 and γ-H2A.X expression in tumor grafts. Figure S12. DHA@MIL-101 induced prominent apoptosis and necrosis of cancer cells in tumor grafts. Figure S13. DHA@MIL-101 promoted the expression of Bax, caspase-3, decreased expression of PCNA in tumor grafts. Figure S14. The body weight of LLC-bearing mice varied little after DHA@MIL-101 treatment. Figure S15. No evident organ toxicity in DHA@MIL-101-treated mice. Figure S16. LPO in LLC was detected using C11-BODIPY probe with confocal microscopy. Figure S17. Quantitative gray analysis of WB results in Fig. 2 to Fig. 4. [file 12951_2022_1455_MOESM1_ESM.doc]

A nanoreactor boosts chemodynamic therapy and ferroptosis for synergistic cancer therapy using molecular amplifier dihydroartemisinin

Xiao-Xin Yang ^3, #^, Xiang Xu ^1,2,#^, Mei-Fang Wang ^1,2^, Hua-Zhen Xu ^5^, Xing-Chun Peng ^1,2^, Ning Han ^1,2^, Ting-Ting Yu ^1,2^, Liu-Gen Li ^1,2^, Qi-Rui Li ^1,2^, Xiao Chen ^5^, Yu Wen ^4,*^, Tong-Fei Li ^1,2,*^

1 School of Basic Medical Sciences, Hubei University of Medicine, Renmin road No. 30, Shiyan, Hubei, 442000, China.

2 Hubei Key Laboratory of Embryonic Stem Cell Research, Taihe hospital of Shiyan, Hubei University of Medicine, Renmin road No. 30, Shiyan, Hubei, 442000, China.

3 School Institute of Chemical Biology and Nanomedicine, State Key Laboratory of Chemo/Biosensing and Chemometrics, College of Chemistry and Chemical Engineering, Hunan University, Changsha, Hunan 410082, China.

4 School of Materials Science and Engineering, Central South University, Changsha, Hunan 410083, China.

5 Department of Pharmacology, School of Basic Medical Sciences, Wuhan University, Donghu Avenue No.185, Wuhan 430072, China; Hubei Provincial Key Laboratory of Developmentally Originated Disease, Wuhan 430071, China.

* E-mail address: [litongfeihappy@163.com](mailto:Litongfeihappy@163.com) (Tong-Fei Li), [yxxawy@csu.edu.cn](mailto:yxxawy@csu.edu.cn) (Yu Wen)

^#^These authors contribute equally to this article

**Supporting information**


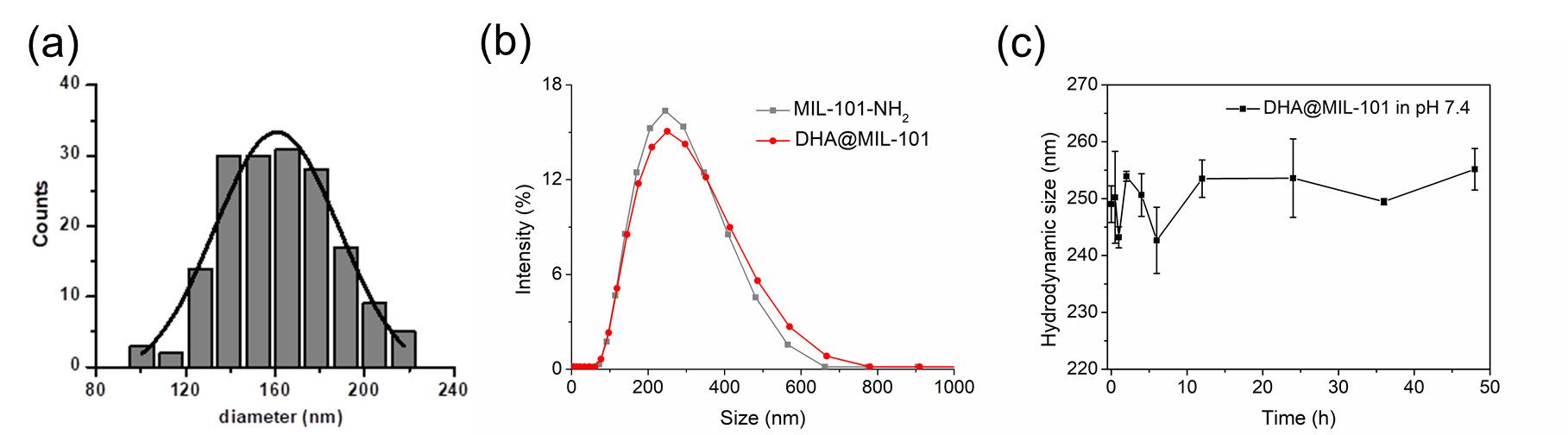


**Figure S1.** (a) Size distribution of MIL-101-NH_2_, (b) hydrodynamic diameters of DHA@MIL-101, (c) the stability evaluation of DHA@MIL-101.





**Figure S2.** UV–vis absorption spectra of DHA, MIL-101-NH2 and DHA@MIL-101-NH2.





**Figure S3.** TGA curves of MIL-101-NH2 and DHA@MIL-101-NH2.


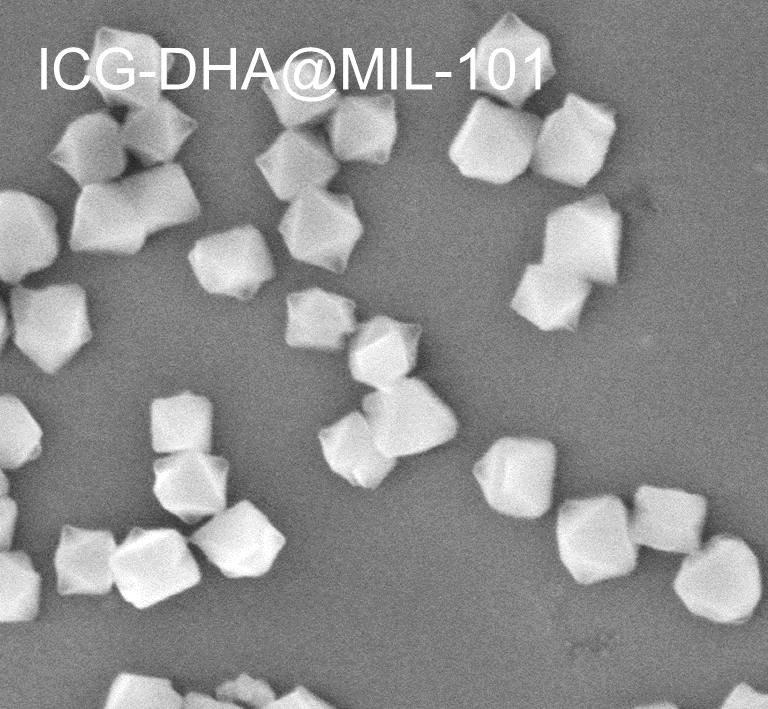


**Figure S4.** SEM images of ICN-DHA@MIL-101.

**Figure S5.** UV–vis absorption spectra of ICG and ICG-DHA@MIL-101-NH2.


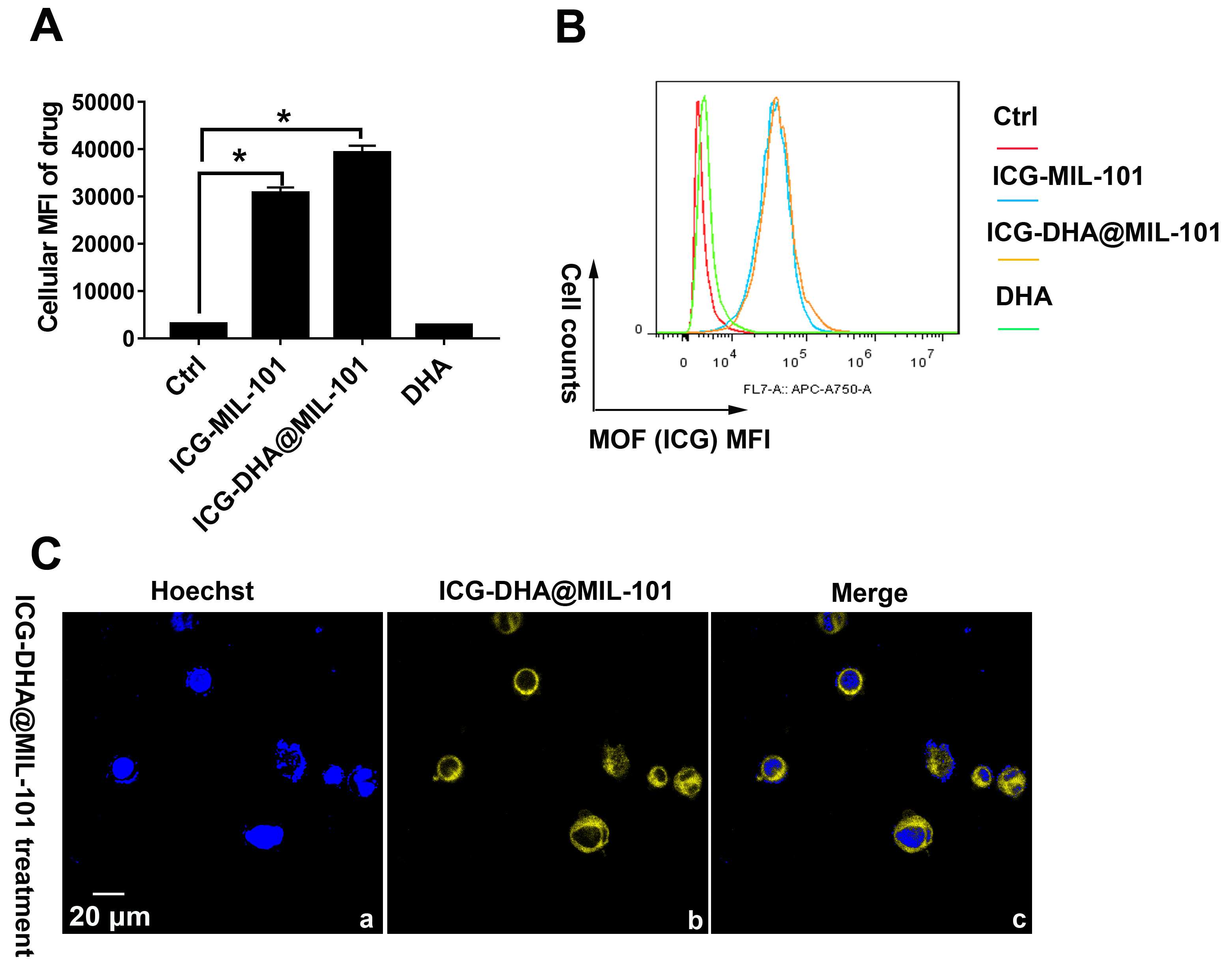


**Figure S6** (A-B) LLC were incubated with the DHA, ICG-MIL-101 and ICG-DHA@MIL-101 (normalized to 8 μg/mL of DHA) for 24 h and cellular fluorescence was measured with flow cytometry (flow cytometry), (C) and confocal microscopy.


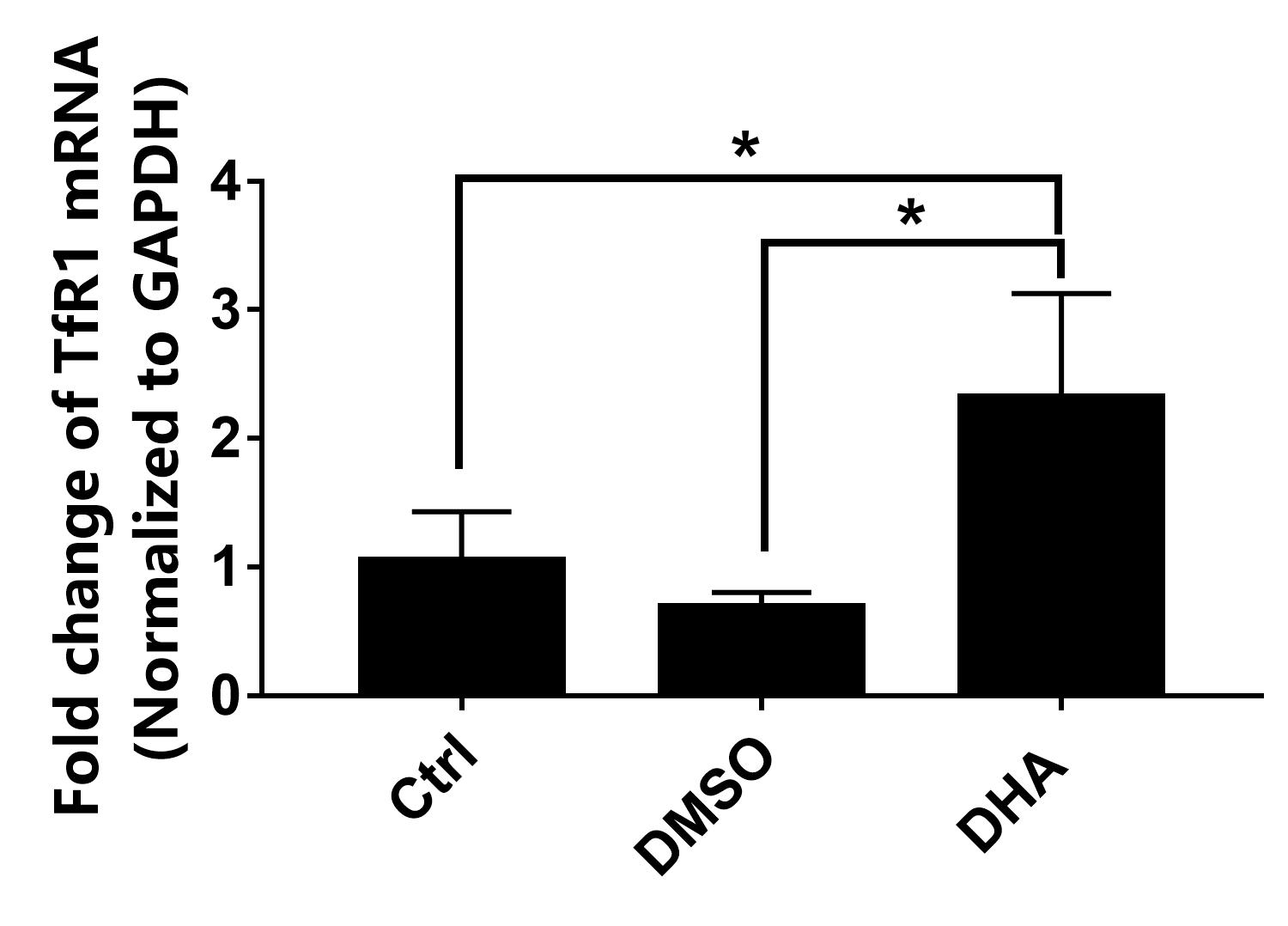


**Figure S7.** DHA up-regulated the expression of TfR1 in LLC. The TfR1 expression was detected through RT-PCR.

**
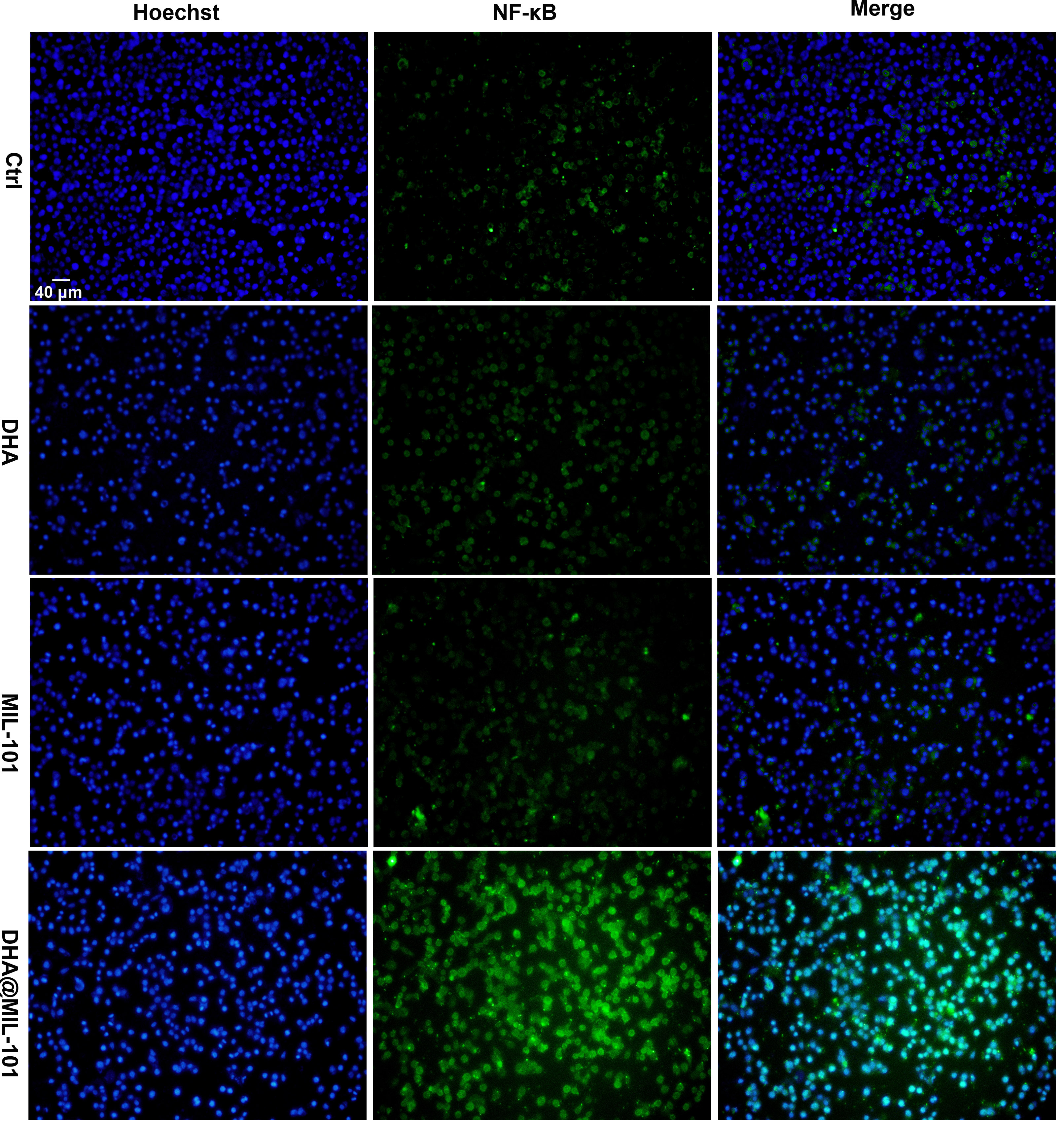
**

**Figure S8.** DHA@MIL-101 promoted Nuclear translocation of NF-κB of LLC, which was assayed by immunofluorescent staining in Figure 3F.

**
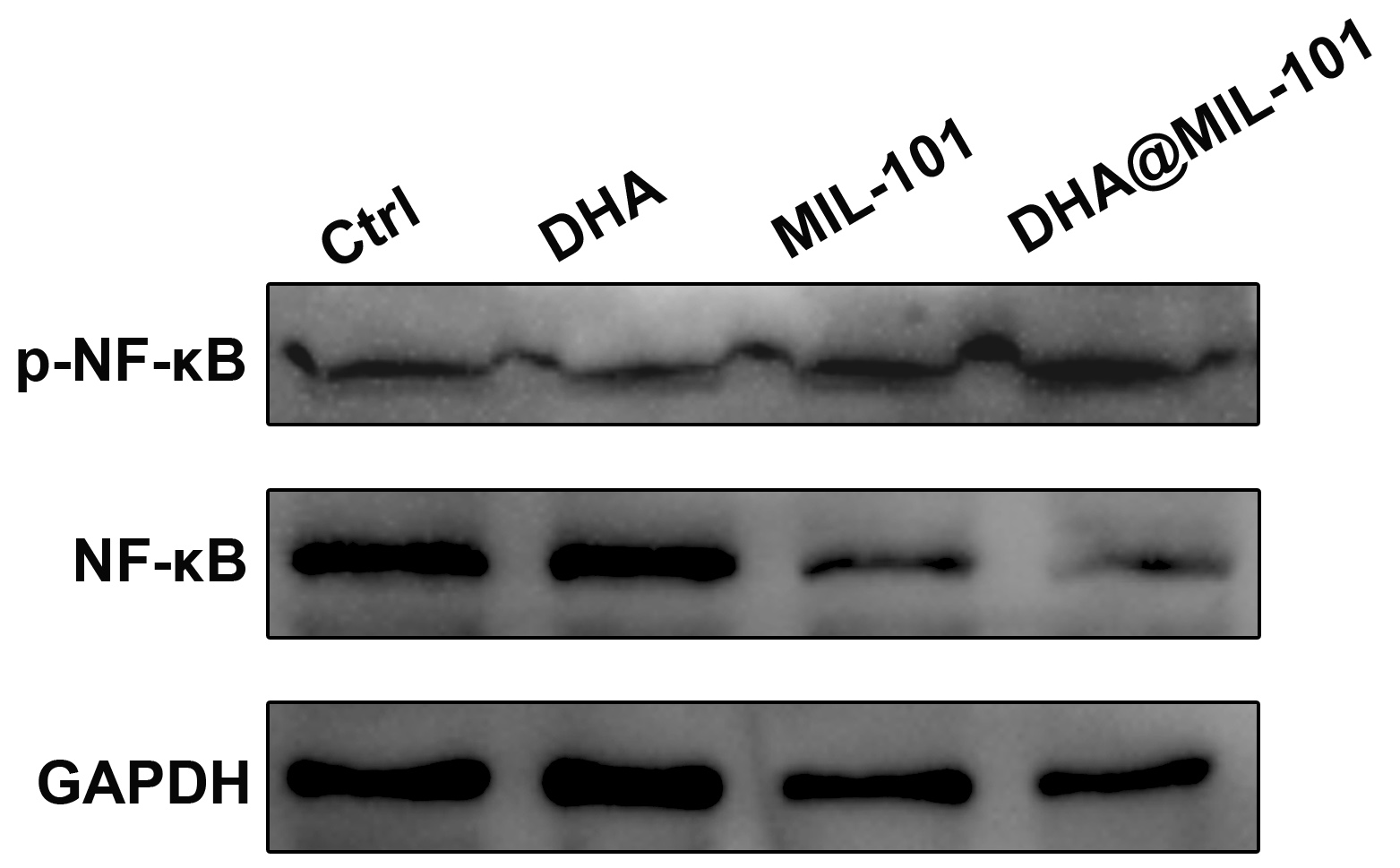
**

**Figure S9.** DHA@MIL-101 promoted phosphorylation of NF-κB of LLC, which was detected by Western blot.

**
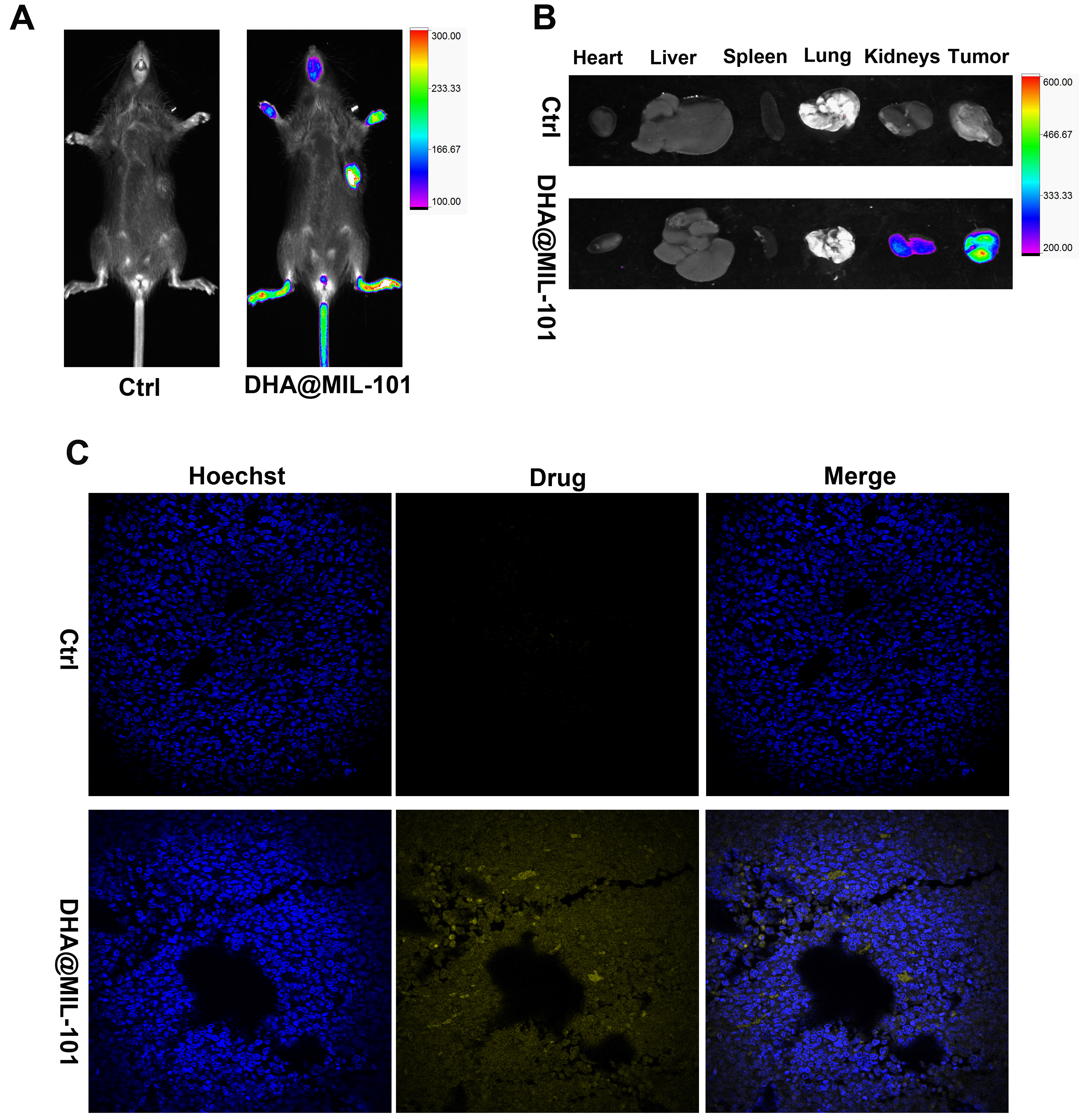
**

**Figure S10.** The ICG-DHA@MIL-101 was distributed in tumor grafts of LLC-bearing mice analyzed using in vivo and organ imaging and confocal microscopy of tissue sections.

**
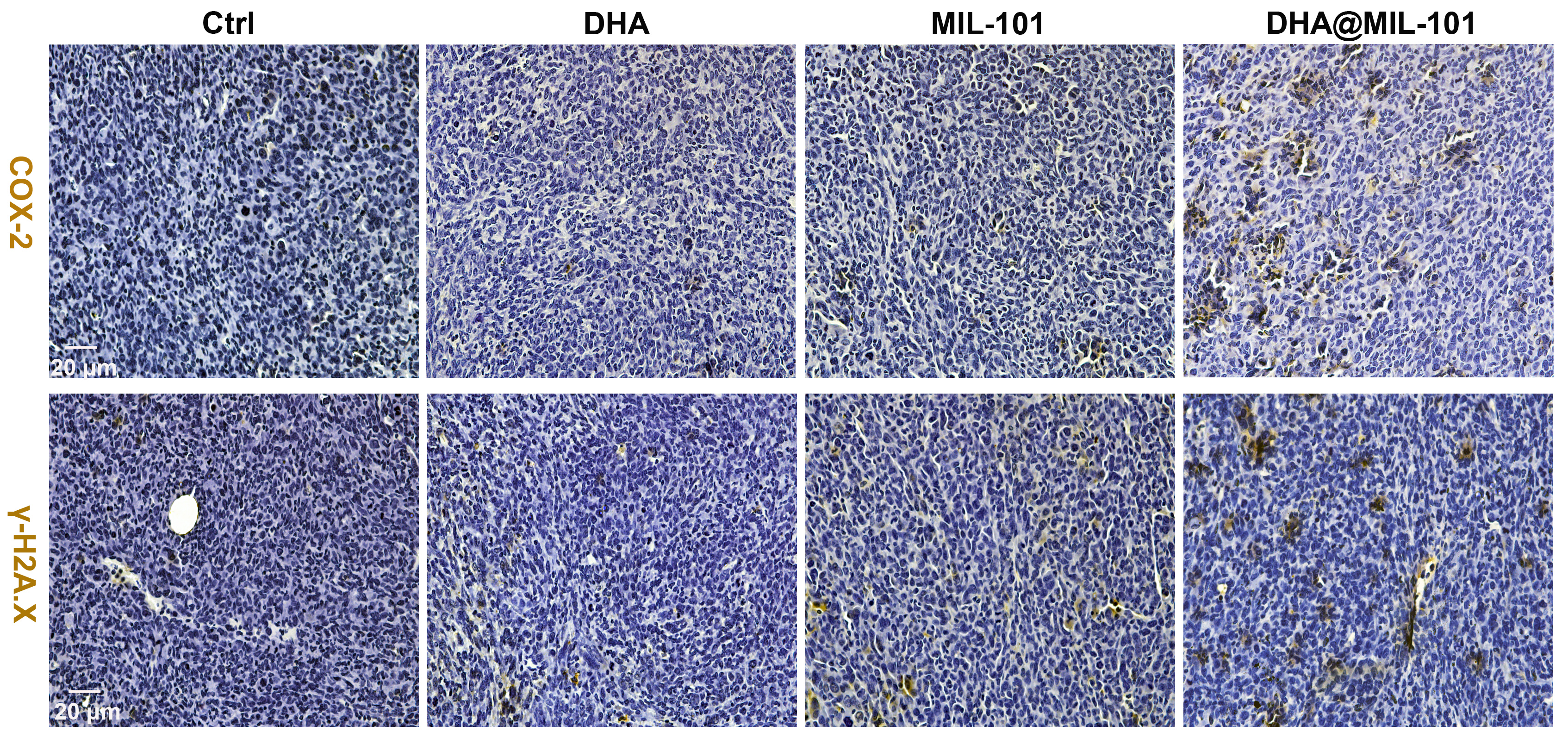
**

**Figure S11.** The tumor grafts of LLC-bearing mice received DHA@MIL-101 treatment showed enhanced expression of COX-2 (biomarkers of ferroptosis) and γ-H2A.X (biomarkers of DNA damage), which was detected by IHC staining.

**
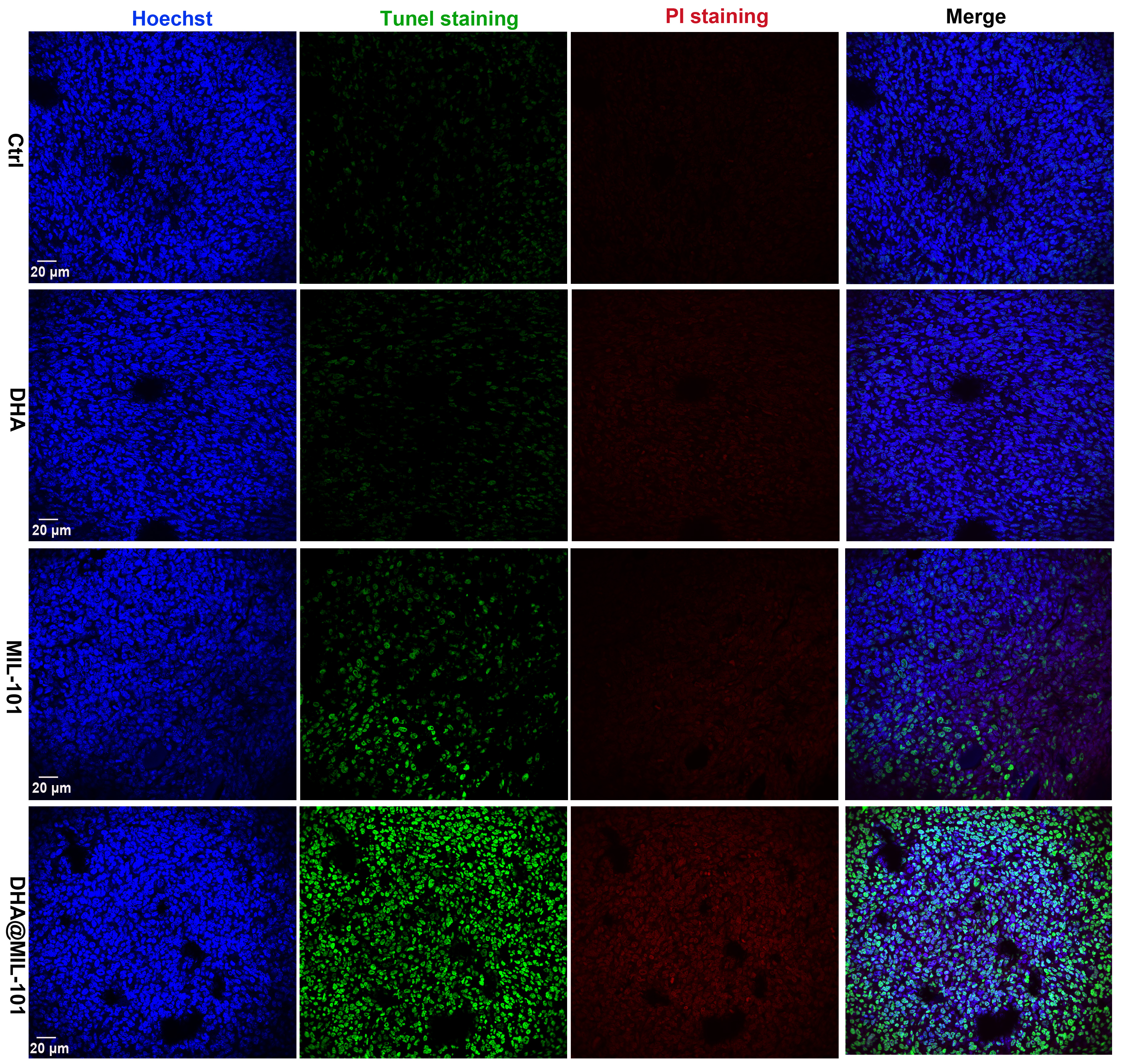
**

**Figure S12.** DHA@MIL-101 induced prominent apoptosis and necrosis of cancer cells in tumor grafts (Figure 5D).

**
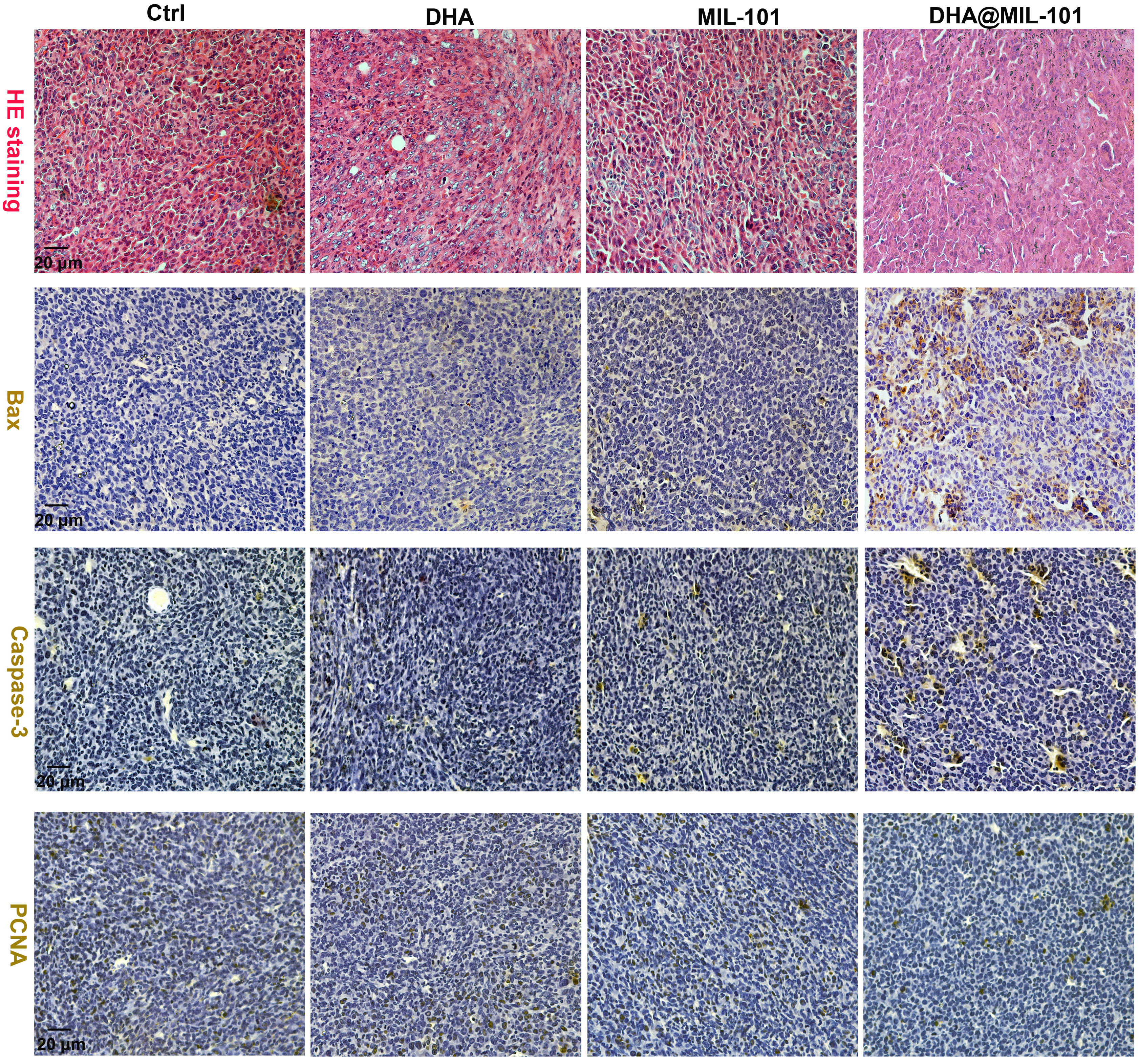
**

**Figure S13.** DHA@MIL-101 promoted the expression of Bax, caspase-3 measured by IHC, which suggested increased apoptosis. In addition, decreased expression of PCNA was detected in mice treated with DHA@MIL-101, indicating inhibition of proliferation of tumor cells.


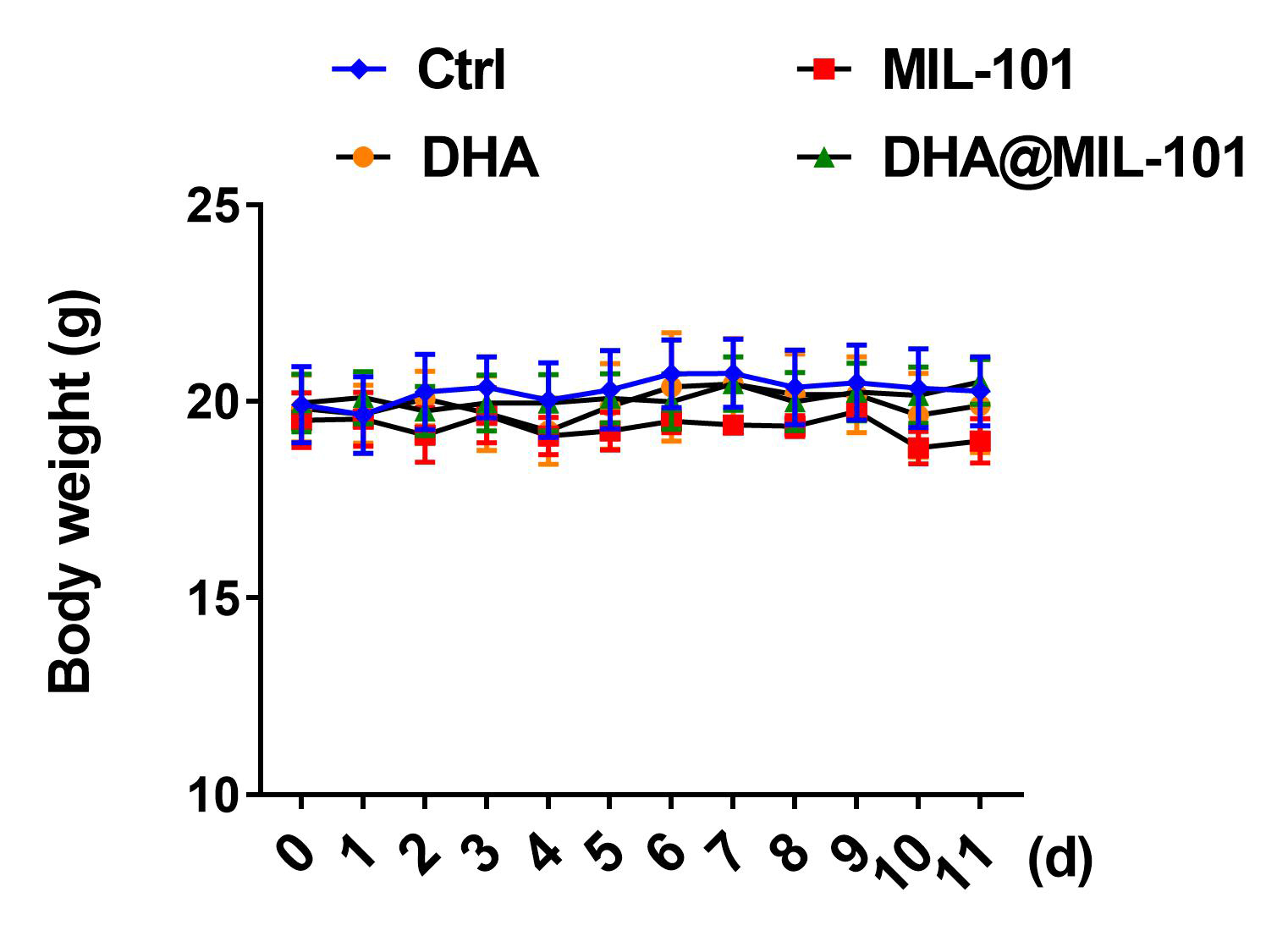


**Figure S14.** The body weight of LLC-bearing mice varied little after DHA@MIL-101 treatment.


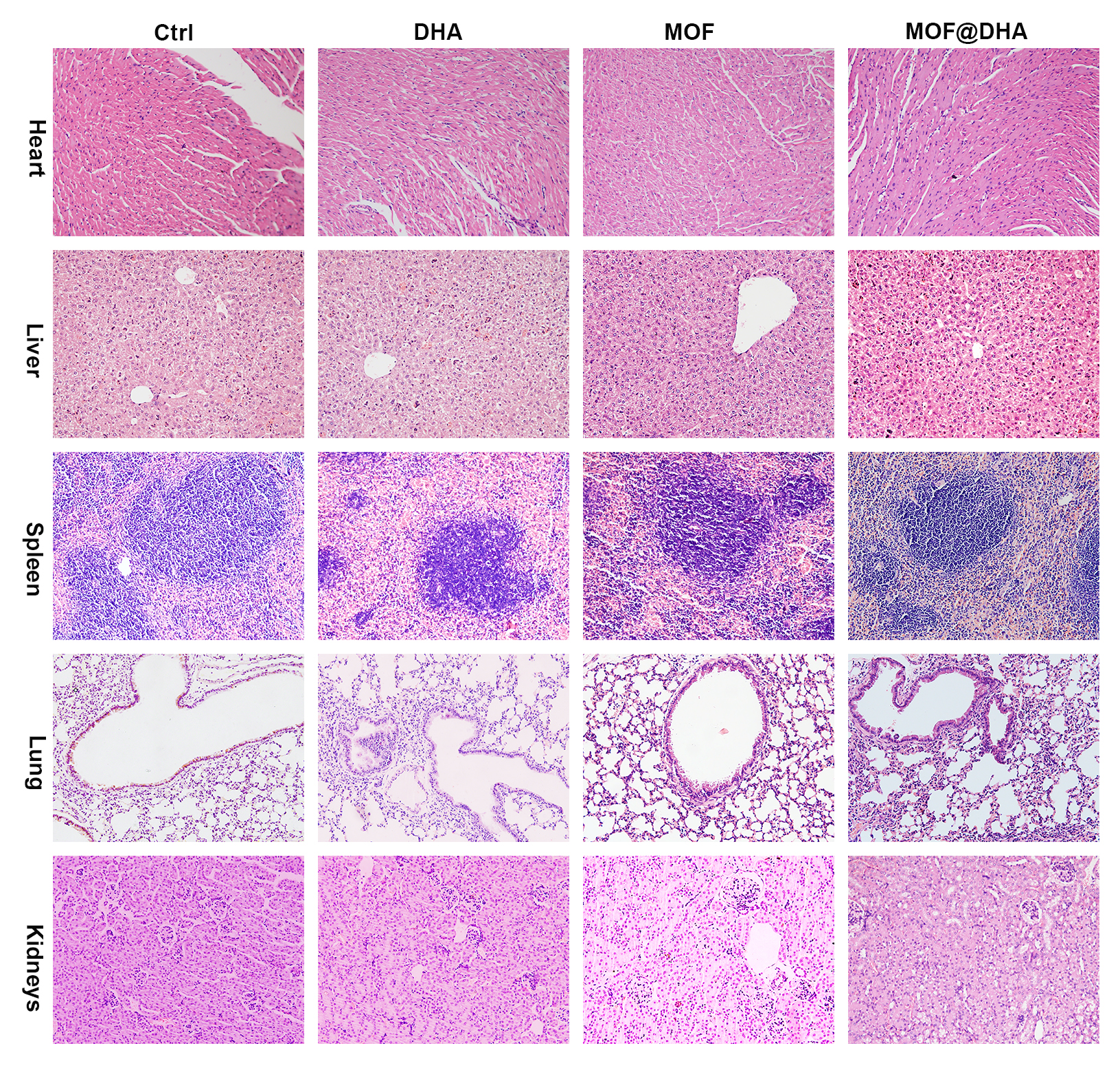


**Figure S15.** No evident organ toxicity in DHA@MIL-101-treated mice. After the treatment, the mice were then sacrificed. HE staining was performed to detect the morphology of vital organs.


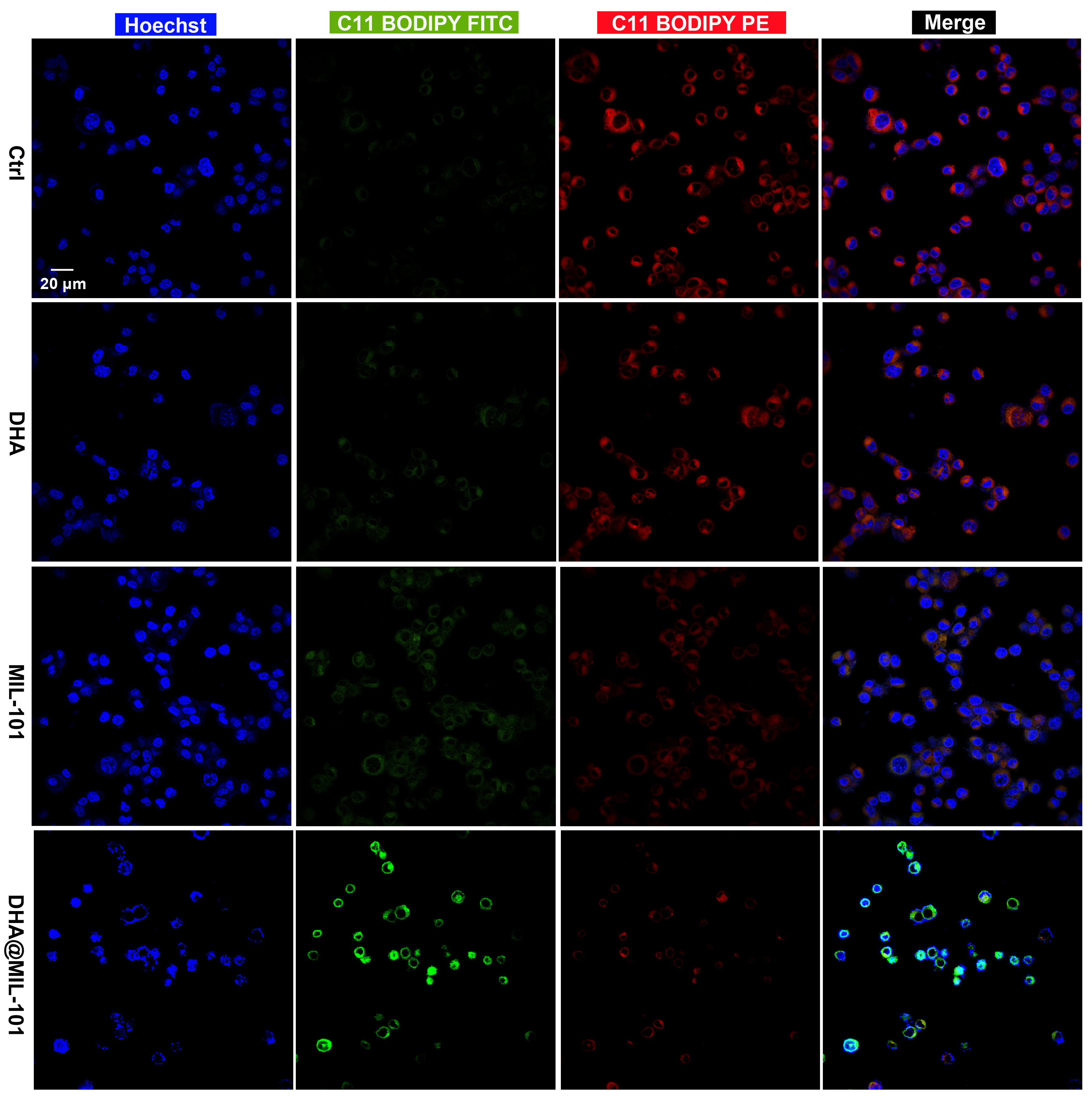


**Figure S16.** Lipid peroxidation (LPO) in LLC was detected using C11-BODIPY probe with confocal microscopy in Figure 2M.


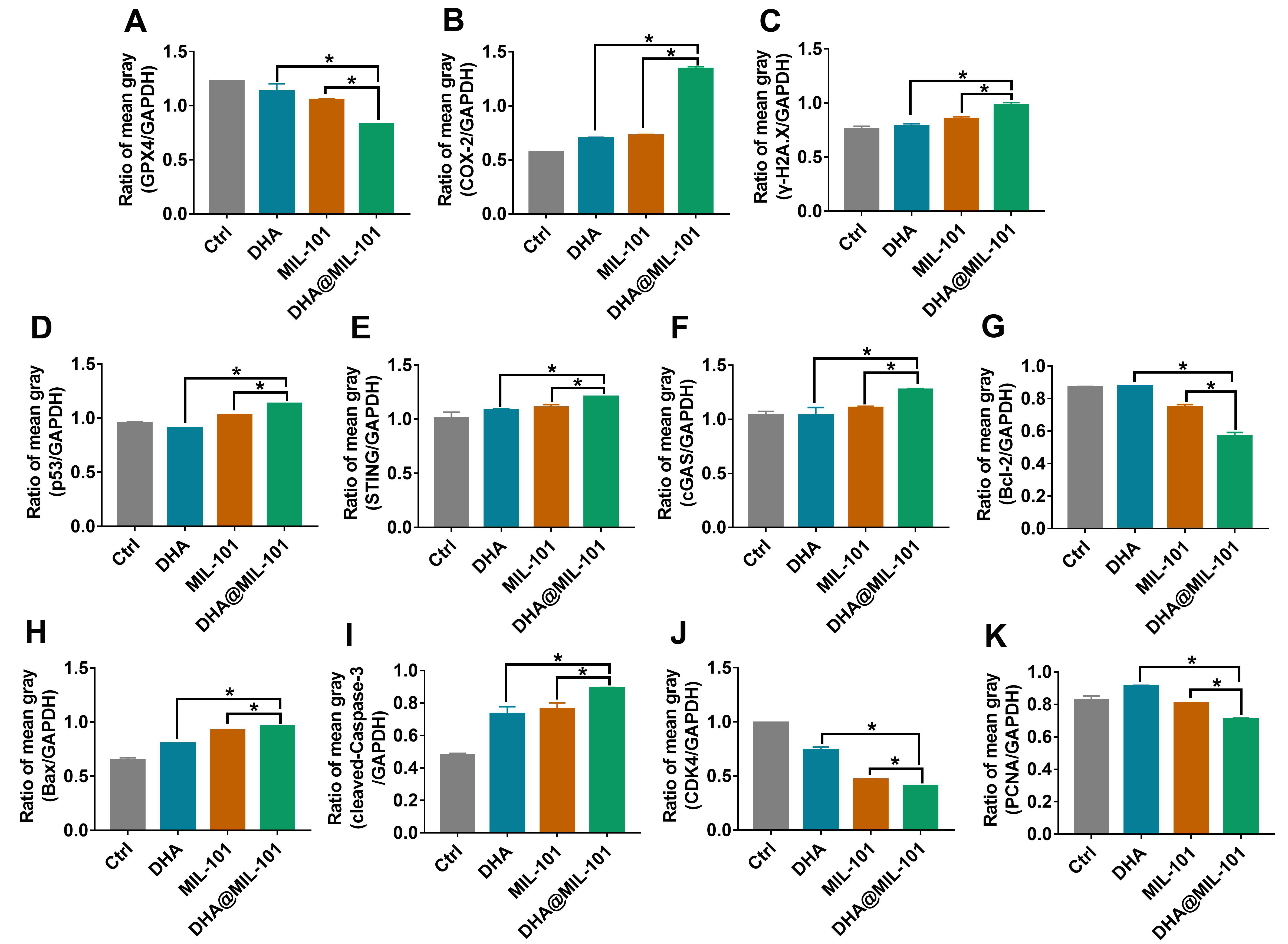


**Figure S17.** Western blotting results in Figure 2-4 were quantitatively analyzed using mean gray. All values are means ± SD (*n* = 3, * *p* < 0.05).
